# Synthesis and Study of Janus-Dione-Based Compounds for Ternary Organic Solar Cells

**DOI:** 10.3390/ma19030533

**Published:** 2026-01-29

**Authors:** Armands Ruduss, Anastasija Rizkova, Fatima Zohra Boudjenane, Elizabete Praulina, Kaspars Traskovskis, Raitis Grzibovskis

**Affiliations:** 1 Faculty of Materials Science and Applied Chemistry, Riga Technical University, 3/7 Paula Valdena Street, LV-1048 Riga, Latvia; armands.ruduss@rtu.lv (A.R.); anastasija.rizkova@rtu.lv (A.R.); fatima-zohra.boudjenane@rtu.lv (F.Z.B.); kaspars.traskovskis@rtu.lv (K.T.); 2 Institute of Solid State Physics, University of Latvia, 8 Kengaraga Street, LV-1063 Riga, Latvia; elizabete.praulina@cfi.lu.lv

**Keywords:** ternary organic solar cells, Janus-dione derivative, synthesis, power conversion efficiency, D-A-D chromophore, non-fullerene acceptor, energy levels

## Abstract

The efficiency of organic solar cells is constantly improving thanks to more advanced materials. Electron donor polymers, such as **PM6** and its derivatives, as well as non-fullerene acceptors (NFAs) **Y6** and **ITIC** and their derivatives, have become the standard materials for organic solar cell studies. To broaden the absorption range of solar cells, so-called ternary organic solar cells have been developed, which add a third material to the active layer. In this work, two chromophores based on the derivatives of the Janus-dione (*s*-indacene-1,3,5,7(2*H*,6*H*)-tetraone) central acceptor fragment, namely **TIIC-1** and **TIIC-2**, were synthesized. Materials were characterized using theoretical and experimental methods, including UV-Vis absorption measurements, cyclic voltammetry, photoemission yield spectroscopy, and photoconductivity. The materials were incorporated as ternary components in **PM6:Y7** bulk heterojunction solar cells. The power conversion efficiency (PCE) of **PM6:Y7:TIIC-1** ternary solar cells was improved compared to binary **PM6:Y7** reference cells. The PCE increased from 11.9% in binary blends to 12.5% in ternary cells. This increase is attributed to the cascade-like energy level arrangement, which facilitates charge transfer in the photoactive layer.

## 1. Introduction

Solar energy is considered one of the essential sources of renewable energy for the transition from a fossil fuel-driven economy to a more sustainable green economy. The recent surge in solar electricity generation has paved the way for solar energy to become a significant factor in global energy markets [1,2]. Currently, the photovoltaics industry is dominated by silicon-based solar cells [3]. However, crystalline silicon solar cells are bulky, rigid, and opaque. Therefore, integrating silicon solar cells into the design of irregularly shaped structures or transparent objects is challenging [4]. Organic solar cells (OSCs) have been proposed as an alternative to established silicon-based photovoltaics. OSCs can be made semi-transparent and flexible enough to be placed where existing cells cannot, such as in the windows of buildings [5], vehicle body parts [6], and even textiles [7]. Additionally, the solution-processability of OSCs enables cheaper, less energy-intensive fabrication methods than those of their silicon-based counterparts [8,9].

Due to the relatively straightforward active-layer manufacturing process, the bulk heterojunction (BHJ) has been widely accepted as one of the most promising OSC technologies. In a BHJ solar cell, exciton generation and splitting take place in an active layer comprising a solid-state mixture of donor (D) and acceptor (A) materials. Donor–acceptor phase separation into a nanostructured bicontinuous interpenetrating network is crucial for achieving the maximum external quantum efficiency (EQE) in BHJ solar cells [10]. The availability of various polymeric and small-molecule donor materials has been well documented [11]. Still, until recently, the range of high-performance acceptor materials was limited to multiple fullerene derivatives [12]. However, the intrinsically poor light-absorption capacity and limited tunability of fullerene derivatives’ energy levels have limited the attainable photocurrent and photovoltage [13]. Recently, the introduction of low-molecular-weight acceptors has provided opportunities for the development of viable alternatives to fullerene-based acceptor materials.

In contrast to fullerenes, the optical properties, electronic energy levels, and morphological properties of NFAs can be readily tuned by modifying their molecular structures. As a result, through continuous improvements in molecular design, NFAs have been developed with expanded light absorption to increase short-circuit current density (*J_SC_*), and with fine-tuned electronic levels to attain maximum open-circuit voltage (*V*_OC_) [14]. Additionally, the morphological properties of NFAs have been extensively studied to control aggregation and ensure optimal phase separation in the active layer [15]. These recent advances have led to the introduction of the first **ITIC** [16] and later **Y6** [17] as the central molecular structure motifs in the rapidly expanding library of NFA materials. Consequently, OSCs utilizing **Y6**-type NFAs are approaching the benchmark power conversion efficiency (PCE) of 20% [18,19,20].

Despite achieving remarkably high PCEs, there is still room for improvement. Extension of the light-absorption window has been proposed as a viable approach to increasing OSC *J_SC_*. Based on this, tandem solar cells have been developed as a possible workaround. A tandem cell consists of two stacked sub-cells with complementary absorption spectra, which are connected via interfacial layers. However, the multilayered structure of tandem cells severely complicates the manufacturing process [21,22]. Ternary organic solar cells (TOSCs) are considered a much simpler option for broadening the light absorption range. In the active layer of TOSCs, either an additional donor or an acceptor material is introduced into the binary BHJ blend. As a result, a ternary D_1_:D_2_:A or D:A_1_:A_2_ system is obtained [23]. In addition to expanding the absorption spectral range, the third component could be used to improve film morphology [24], adjust energy levels [25], and facilitate effective charge transfer [26].

**Figure 1:**
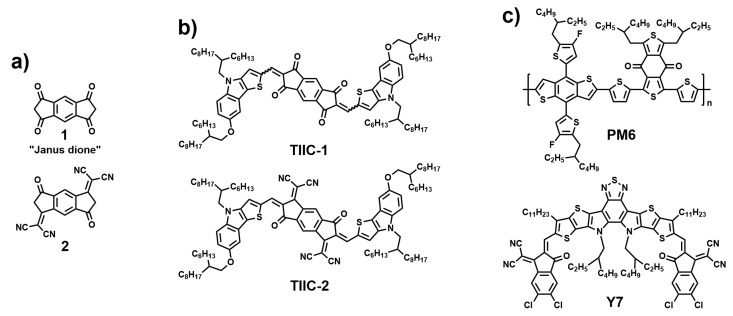
(**a**) Structure of the “Janus-dione” acceptor fragment **1** and its dicyanomethylene substituted analog **2**; (**b**) structures of the explored ternary-blend third-component chromophores **TIIC-1** and **TIIC-2**; (**c**) structure of the donor material **PM6** and NFA material **Y7**.

Recently, our research team investigated a group of D-π-A-π-D type chromophores with a central acceptor fragment composed of *s*-indacene-1,3,5,7(2*H*,6*H*)-tetraone **1** (so-called Janus-dione) and its derivative **2**, where two of the four carbonyls are substituted with dicyanomethylene groups (see Figure 1a) [27]. We showed that Janus-dione-based dyes exhibit excellent light-absorption properties, prompting us to investigate their possible application as the third component in ternary solar cells. In this paper, we report the synthesis of two Janus-dione-derived chromophores, **TIIC-1** and **TIIC-2** (see Figure 1b). In these compounds, the Janus-type electron-accepting building blocks **1** and **2** are bound to electron-donating 4*H*-thieno [3,2-*b*]indole groups. Due to its electron-rich, planar, π-conjugated structure, thienoindole has been widely used as a donor fragment in organic electronics [28,29,30,31]. The planarity of the thienoindole unit is a direct consequence of its fused, aromatic structure. This structural rigidity and high degree of planarity provide advantages for photovoltaic performance. The flat surface of the thienoindole allows molecules to pack tightly in a “face-on” orientation with respect to one another. This reduces the π-stacking distance, thereby significantly facilitating inter-chain charge hopping and increasing charge mobility [32,33]. Additionally, the straightforward synthesis of thienoindole derivatives allows structural modifications, such as the attachment of various solubilizing alkyl groups [34]. The UV-Vis absorption spectra and energy-level structures of the synthesized materials, both in solution and in films, were characterized. The studied compounds do not show high efficiency as standalone NFAs in OSCs. However, incorporating **TIIC-1** as the third component in a commonly used **PM6:Y7** [35,36,37,38] (see Figure 1c) active-layer blend increases *J*_SC_ and PCE.

## 2. Materials and Methods

### 2.1. Materials

The reagents and solvents used for the synthesis of the target compounds **TIIC-1** and **TIIC-2** were purchased from commercial suppliers (Sigma Aldrich (St. Louis, MO, USA), Fluorochem (Hadfield, UK), Alfa Aesar (Ward Hill, MA, USA), etc.). The solvents were dried according to standard methods. DMF and 1,2-dichlorobenzene were distilled from CaH_2_ in a vacuum before use. The exact synthetic procedures are given in the Supplementary Materials. Nuclear magnetic resonance (NMR) spectra were obtained on a Bruker Avance 500 MHz spectrometer (Billerica, MA, USA). CDCl_3_ residual signals were used as an internal reference (^1^H, δ 7.26; ^13^C, δ 77.16) for the NMR spectra.

### 2.2. Computational Methods

Q-Chem 6.2.2 software was used to perform density functional theory (DFT) geometry optimizations and time-dependent DFT (TD-DFT) calculations for excited-state energy prediction. Geometry optimization was performed using the wB97X-D functional and the def2-SVP basis set. A polarizable continuum model (PCM) was used with the PBE0/def2-TZVP theory level to calculate TD-DFT in dichloromethane (dielectric constant 8.9). The highest occupied molecular orbital (HOMO), lowest unoccupied molecular orbital (LUMO), natural transition orbitals (NTOs), and electrostatic potential (ESP) surfaces were visualized in the IQmol 3.1 program. HOMO-LUMO overlap integrals were calculated using Multiwfn 3.8 software [39].

### 2.3. Sample Preparation and Measurement Systems

UV−Vis absorption measurements in solution were carried out at a concentration of 10^−5^ M in dichloromethane. The thin-film samples for UV-Vis absorption measurements were prepared by spin-coating from chloroform solutions. UV-Vis spectra were obtained using a Perkin Elmer Lambda 650 (Waltham, MA, USA) spectrometer.

Cyclic voltammetry measurements were performed with a PARSTAT 2273 (Princeton Applied Research, Oak Ridge, TN, USA) potentiostat. A 0.1 M TBAF solution in anhydrous dichloromethane was used as the supporting electrolyte. The electrochemical redox reactions were examined under an Ar atmosphere. The measurements were carried out in a three-electrode cell configuration with a stationary glassy carbon disk (Ø 0.5 cm) as the working electrode, a Pt wire as the auxiliary electrode, and a silver wire as the pseudoreference electrode. Ferrocene/ferrocenium (Fc/Fc^+^) couple was used as the internal reference, and all the potentials were calibrated against Fc/Fc^+^. Peak onset potentials (referenced against Fc/Fc^+^) were used for the determination of *E*_ox_ and *E*_red_.

Indium tin oxide (ITO)-coated glass substrates with a sheet resistance of 20 Ω/sq were used for the photoelectrical measurements. The substrates were cleaned by ultrasonification in chloroform and acetone, followed by rinsing in deionized water. Afterwards, they were washed with a 2% detergent solution, rinsed in deionized water, and then ultrasonicated in isopropyl alcohol, where they were kept until used to make samples. The samples for energy level determination were made by dissolving the studied materials in chlorobenzene at a concentration of 10 mg/mL. The film was obtained by spin-coating using the following parameters: a rotation speed of 800 rpm, acceleration of 800 rpm/s, and a rotation time of 60 s. Afterwards, the samples were dried on a hotplate at 120 °C for 10–15 min.

The ionization energy level values of the studied materials were obtained using a self-built photoemission yield spectroscopy (PYS) system consisting of an ENERGETIQ Laser-Driven Light Source (LDLS EQ-99, Energetiq Technology Inc., Wilmington, MA, USA) white-light source, a Spectral Products DK240 1/4 m (Putnam, CT, USA) diffraction grating monochromator, and a Keithley 617 electrometer (Keithley Instruments, Cleveland, OH, USA). The measurements were carried out in a vacuum at approximately 1 × 10^−5^ mbar. The spectral range of the measurements was 4.5–6.8 eV, with a step size of 0.05 eV. The electrometer was used to measure the photoemission current and to apply a 50 V bias between the sample and the electrode. The distance between the sample and the electrode was around 2 cm. The energy gap between the ionization energy level and electron affinity level was obtained from photoconductivity measurements using the same equipment as for the PYS measurements. Here, the electrical contacts were connected to the organic material film. The spectral range of the measurements was 930–450 nm, with a step size of 10 nm.

The solar cells were made by initially dissolving the organic materials (**PM6**, **Y7**, **TIIC-1**, and **TIIC-2**) in chloroform to obtain a 10 mg/mL solution. The electron transport material PDINO was dissolved in methanol at a concentration of 1.5 mg/mL. The vials containing the solutions were placed on a hotplate at 50 °C, and magnetic stirring was turned on. After 30 min, when the organic materials were completely dissolved, the solutions were mixed to obtain the desired mass ratio of organic materials. For the reference cell, the **PM6:Y7** mass ratio was 10:10. Previously cleaned ITO-coated substrates were cleaned in the UV-ozone generator for at least 10 min. The PEDOT:PSS (Al4083, Clevios™, Heraeus Epurio, Leverkusen, Germany) layer was deposited by spin-coating. The rotation speed was 2000 rpm, the acceleration was 2000 rpm/s, and the rotation time was 60 s. Afterwards, the substrates were dried in air on a hotplate at 150 °C for 15 min. The active layer was spin-coated in the argon-filled glovebox. The spin-coating parameters were as follows: rotation speed, 800 rpm; acceleration, 800 rpm/s; and rotation time, 60 s. The covered substrates were dried on a hotplate at 110 °C for 10 min. Then the electron transport layer (PDINO) was deposited by spin-coating with the following parameters: rotation speed of 3000 rpm, acceleration of 3000 rpm/s, and rotation time of 60 s. The samples were dried on a hotplate at 110 °C for 2 min. Then the samples were transferred to the glovebox, where a Moorfield Nanotechnology/Jacomex MiniLab LT090A-MX/GP (Concept)-T thermal evaporator (Moorfield Nanotechnology, Knutsford, UK) was used to deposit 100 nm thick silver (Ag) electrodes. It was performed at a pressure of 4 × 10^−6^ mbar with a speed of 0.2 nm/s. Samples with a 4 × 4 mm^2^ pixel size were obtained using shadow masks. Solar cells were characterized using a ScienceTech SS150 (London, ON, Canada) solar simulator with a light intensity of 100 mW/cm^2^ and a standard AM 1.5G filter. The current-voltage characteristics were measured using a Keithley 6517B electrometer (Keithley Instruments, Cleveland, OH, USA) in the range of −0.10 V to 0.90 V with a 10 mV step. From these curves, the main parameters for solar cell characterization were obtained: short-circuit current density (*J*_SC_), open-circuit voltage (*V*_OC_), fill factor (FF), and PCE. The EQE measurements of the solar cells were performed in the same self-built system used for photoconductivity and PYS measurements. The measurements were performed over the spectral range of 1000–400 nm with a step size of 10 nm.

## 3. Results

### 3.1. Synthesis

**Figure 2:**
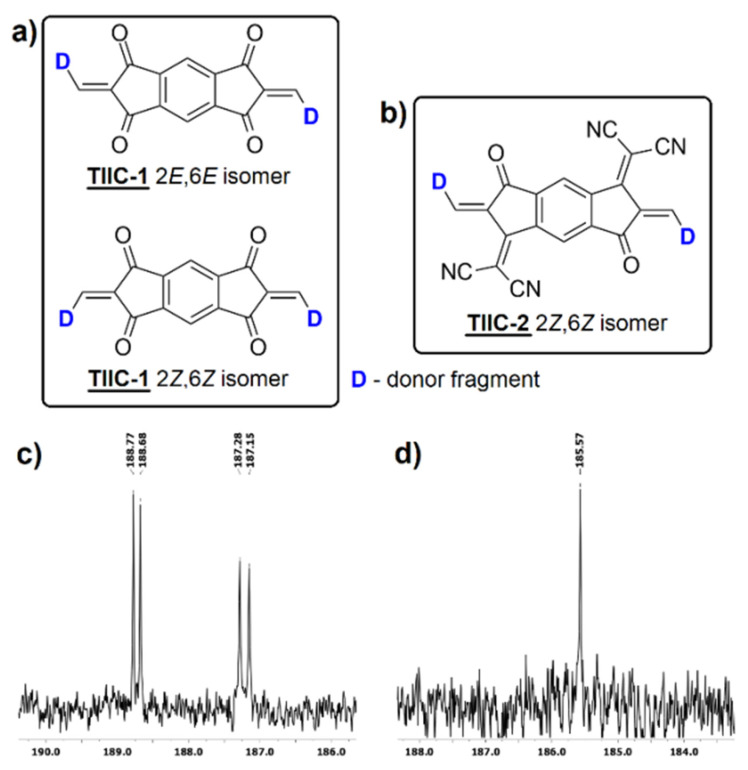
(**a**,**b**) Observed isomers at the double bond of the central acceptor fragment of **TIIC-1** and **TIIC-2**, respectively; (**c**,**d**) ^13^C-NMR signals of the indacene carbonyl C atoms of **TIIC-1** and **TIIC-2**, respectively.

The synthesis of target compounds **TIIC-1** and **TIIC-2** was carried out via Knoevenagel condensation reaction between an aldehyde group containing a thieno[3,2-*b*]indole-based electron-donating fragment and Janus-dione-based electron acceptors **1** and **2**. In the case of **TIIC-1,** the condensation reaction proceeded under heating in pure acetic anhydride. At the same time, the synthesis of **TIIC-2** was carried out in acetonitrile, and acetic anhydride was added in a catalytic amount. Both products were obtained in moderate yields of 44% and 42%, respectively. Analysis of the ^13^C NMR spectra of **TIIC-1** revealed splitting of the corresponding *s*-indacene ^13^C NMR signals. It has been shown in the literature [27,40] that condensation of Janus-dione (**1**) with an aromatic aldehyde yields a mixture of *E*,*E* and *Z*,*Z* isomers (see Figure 2a). The similar relative intensities of the corresponding ^13^C NMR signals (see Figure 2c) indicate the formation of a 1:1 mixture of the isomers.

A thorough examination of the ^1^H NMR spectra of **TIIC-1** revealed that instead of the expected 12 aromatic protons, 10 aromatic protons are observed. A more detailed analysis of the spectra revealed that the missing aromatic proton signal could be related to a slight baseline bump observed between approximately 8.4 and 7.4 ppm (Figure S1, Supplementary Materials). To further elaborate, we decided to perform NMR experiments at elevated temperatures. C_2_Cl_4_D_2_ was used as the solvent due to its higher boiling point. A practically identical spectrum for aromatic signals to that of CDCl_3_ was observed in C_2_Cl_4_D_2_ at room temperature (see Figure S2a, Supplementary Materials). However, at an elevated temperature (70 °C; see Figure S2b, Supplementary Materials), an increase in spectral resolution and a slight upfield shift in the signals were observed. Foremost, the very broad signal stretching from approx. 8.4 to 7.4 ppm was rectified to a more pronounced signal at approximately 8.32 ppm. Thus, the two “missing” aromatic proton signals were revealed. The observed changes in the NMR spectrum with varying temperature may be related to the presence of rotamers. As the temperature increases, the rotation rate increases and the coalesced signals narrow. The structure of possible rotational isomers is given in Figure S3. However, it must be noted that the presence of *E*,*E* and *Z*,*Z* isomers complicates the interpretation of ^1^H NMR spectra and impedes an accurate assessment of the conformational structures.

Meanwhile, no signal splitting was observed in the ^13^C NMR spectra of **TIIC-2** (see Figure 2d), indicating the formation of only one isomer. In this case, the steric effect of the dicyanomethylene groups directs the attachment of the donor groups to a strictly *Z*,*Z* configuration (see Figure 2b). Additionally, no distinct rotational isomerism was observed. It has been widely shown that the crystalline properties of the photoactive layer materials influence the morphology of the donor–acceptor blend in BHJ. The presence of a 1:1 *E*,*E* and *Z*,*Z* isomer mixture and distinct rotation isomers could be considered unfavorable for the formation of nanoscale crystalline domains of **TIIC-1**. However, in TOSCs, an alloy model (AM) has been identified as one of the operating mechanisms of the active layer [23]. In the AM, the ternary component (an acceptor or a donor) couples with either the primary acceptor or donor material, forming either an A_1_:A_2_ or D_1_:D_2_ electronic alloy. For the AM, compatibility of the ternary material with either the acceptor or donor material (e.g., good miscibility and well-adjusted energy levels) is more significant than the crystallinity properties of the ternary component as an individual component [25].

### 3.2. Computational Calculations

**Figure 3:**
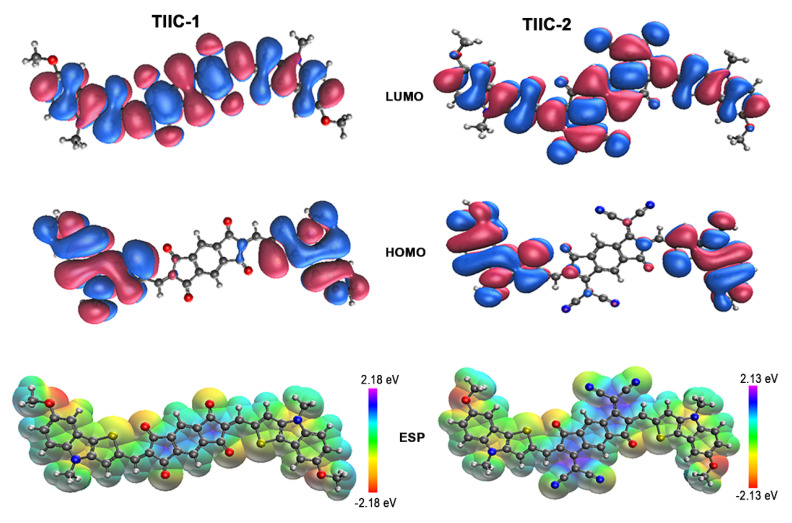
Representations of the calculated frontier molecular orbitals and ESP surfaces for the synthesized compounds **TIIC-1** and **TIIC-2**. Calculations were performed at the wB97X-D/def2-SVP level. The red regions of the map represent areas with high electron density. The blue color represents sites where the molecule is positively charged.

Density functional theory DFT was employed to estimate the molecular geometry of **TIIC-1** and **TIIC-2,** and to conduct prediction of the photophysical properties. Figure 3 shows that the HOMO is localized on the electron-rich 4*H*-thieno[3,2-*b*]indole fragments with similar energies of −5.79 and −5.91 eV for **TIIC-1** and **TIIC-2**, respectively. The LUMO is located on the central indacene fragment, indicating a strong electron-accepting property. In the case of **TIIC-2,** the LUMO energy is 0.46 eV lower than in **TIIC-1** (see Table 1). It is evident from the LUMO energies that the dicyanomethylene-substituted Janus-dione in **TIIC-2** shows more potent electron-accepting properties than the unmodified Janus-dione fragment in **TIIC-1**.

Furthermore, the electrostatic potential (ESP) maps for **TIIC-1** and **TIIC-2** are shown in Figure 3, depicting how electron density is distributed within the molecules, with regions of different colors representing different densities. The red regions of the map represent areas with high electron density. The blue color represents sites where the molecule is positively charged. Additionally, light blue spots have a less electropositive potential than darker blue parts, whereas green areas have a neutral electrostatic potential. The ESP map reveals that the central indacene ring system of both molecules is positively charged. On the contrary, the negative charge is localized peripherally on the nitrogen, oxygen, and sulfur atoms of the thienoindole groups and on the carbonyl and cyano groups attached to the indacene core.

The lower-lying singlet and triplet excited state energies were established through the TD-DFT approach. The charge transfer (CT) between electron-donating thienoindole and electron-deficient indacene fragments, associated with electron transfer between HOMOs and LUMOs, is attributed to S_0_→S_1_ and S_0_→T_1_ transitions. **TIIC-1** has the largest S_1_ and T_1_ energy, which decreases in **TIIC-2** due to the lower LUMO energy. It is noticed that for both investigated compounds, the S_0_→S_3_ transition is more dominant than energetically lower-lying S_0_→S_1_ and S_0_→S_2_ excitations, as indicated by much higher oscillator strength (*f_osc_*) values. As shown by the calculated natural transition orbitals (NTOs) (Figures S4 and S5, Supplementary Materials), the S_1_ and S_2_ states can be attributed to charge-transfer (CT) transitions, in which electron density shifts from the peripheral donor groups to the indacene-centered LUMO. In the case of S_3_, the electrons originate from the HOMO-1 orbital, which is delocalized across the whole π-electron system of the chromophore. The increased overlap in the involved orbitals for the S_0_→S_3_ transition is responsible for the notably increased *f_osc_* value. This result indicates that the light absorbance properties of the investigated compounds are mainly determined by energetically higher-lying singlet states.

### 3.3. UV-Vis Spectroscopy

**Figure 4:**
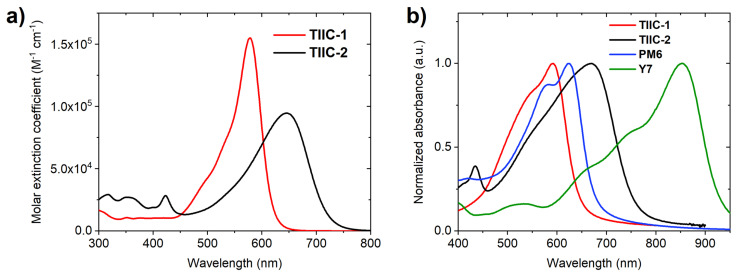
(**a**) UV-Vis absorption spectra of **TIIC-1** and **TIIC-2** in dichloromethane (10^−5^ M); (**b**) UV-Vis absorption spectra of **TIIC-1**, **TIIC-2**, **PM6,** and **Y7** in thin films.

The UV-Vis absorption spectra of the investigated materials in dichloromethane are shown in Figure 4a. The Janus-dione-based chromophore **TIIC-1** exhibits an intense, sharp absorption band with a maximum at 578 nm. The extinction coefficient for this band reaches 1.55 × 10^5^ M^−1^·cm^−1^, and vibrionic features are observable as weakly pronounced shoulders. The introduction of the dicyanomethylene groups in the Janus-dione acceptor fragment of **TIIC-2** leads to a redshift in *λ*_max_ to 646 nm. Simultaneously, a significant broadening of the absorption band and lowering of the extinction coefficient (*ε*_max_ = 0.95 × 10^5^ M^−1^·cm^−1^) are evident. To evaluate the light-harvesting capability of these materials as ternary components in **PM6**/**Y7** blends, thin-film sample absorption measurements were performed (see Figure 4b). Both of the studied compounds show bathochromic shift and broadening of the absorption bands in thin films. The absorption of **TIIC-1** in thin film samples is slightly blue-shifted relative to the absorption of **PM6**. For **TIIC-2,** the absorption band is well-positioned between the absorption maxima of **PM6** and **Y7**.

### 3.4. Cyclic Voltammetry

The electronic energy level structures of **TIIC-1** and **TIIC-2** were studied by cyclic voltammetry (CV) in dichloromethane. The results of the CV measurements are given in Table 2, and the corresponding cyclic voltammograms are provided in the Supplementary Materials (Figure S6). The studied compounds exhibit similar oxidation potentials at approximately 0.6 V. Given that both materials share the same donor fragment, we attribute this transition to the oxidation of the electron-rich thienoindole fragment. In the case of reduction potential, a significantly larger difference is observed. For **TIIC-1**, *E*_red_ is −1.26 V, while **TIIC-2** exhibits an *E*_red_ of −0.82 V. We conclude that reduction is associated with the central indacene-based acceptor fragment, and the introduction of the electron-accepting dicyanomethylene groups leads to a noticeable cathodic shift in reduction potential for **TIIC-2**. The HOMO and LUMO values derived from the electrochemical measurements [41] correlate with the previously discussed DFT calculations.

### 3.5. Energy Level Determination

Because the energy levels of materials are influenced by intermolecular interactions, CV measurements carried out in solution can yield inaccurate electron affinity and ionization energy values. Therefore, photoemission yield spectroscopy (PYS) and photoconductivity measurements were used to determine the ternary OSC energy level structure in thin films.

The ionization energy (*E*_ioniz_) of the studied materials was determined using PYS. The photoemission yield (*Y*(*hν*)) is the number of emitted electrons per incident photon. This yield is proportional to the difference between the photon energy and the ionization energy level of the studied material [42,43,44]:

(1) $$Y\left(h\nu \right)\propto {\left(h\nu -E_{ioniz}\right)}^{5/2}$$

**Figure 5:**
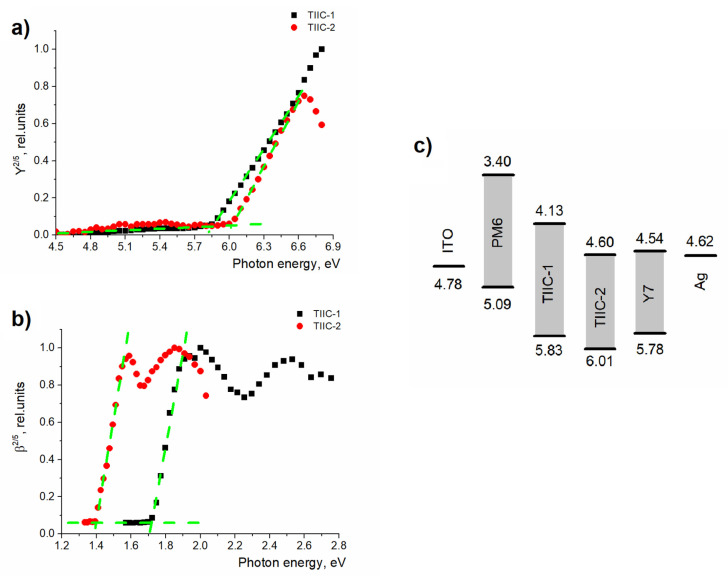
(**a**) Ionization energy level determination. (**b**) Energy gap determination. (**c**) Energy level scheme of the used materials. Green dashed lines are guides for eyes to show linear approximations.

The ionization energy levels of the materials were obtained from *Y*^2/5^(*hν*), the threshold energy at which photoelectron emission increases rapidly (see Figure 5a).

As shown in Figure 5a, the ionization energy of **TIIC-1** is 5.83 ± 0.03 eV, while that of **TIIC-2** is deeper at 6.01 ± 0.03 eV. This is more than 0.2 eV below the ionization energy of **Y7**. These measured values are close to those obtained from the DFT calculations (see Table 1).

The energy gap (*E*_gap_) value of the studied materials was obtained from the spectral dependence of photoconductivity. From these measurements, the photoconductivity efficiency *β*(*hν*) was calculated as the number of generated photoelectrons per incident photon. Similarly to the photoelectron emission (Equation (1)), *β*(*hν*) is proportional to the *E*_gap_ and photon energy [45,46]:

(2) $$\beta \left(h\nu \right)\propto {\left(h\nu -E_{gap}\right)}^{5/2}$$

The photoconductivity efficiency spectral dependence for **TIIC-1** and **TIIC-2** is shown in Figure 5b. From this, the *E*_gap_ for **TIIC-1** was determined to be 1.70 eV. From this, we obtain the **TIIC-1** electron affinity level of 4.13 eV. A combination of a deep ionization energy level and small gap energy (1.41 eV) means that the electron affinity of **TIIC-2** is also relatively deep, at 4.60 eV, which is close to the values of the electron acceptor material **Y7** (electron affinity of 4.54 eV) and the work function of silver (4.62 eV). Figure 5c shows the complete energy level scheme for the materials used in solar cells. As shown, electrons from **PM6** can be transferred to **TIIC-1** and then to **Y7**, creating a cascade effect. **TIIC-2** and **Y7** are most likely working in parallel, without charge transfer between them. Additionally, a photoluminescence quenching experiment examining different ratios of **TIIC-1** and **Y7** in toluene solution was conducted to provide additional evidence for the cascade charge-transfer pathway (see Figure S7, Supplementary Materials). There is effective excitation transfer between the two compounds because the emission of **TIIC-1** is quenched upon the introduction of **Y7** into the mixture.

### 3.6. Solar Cell Characterization

**PM6:Y7** bulk heterojunction solar cells were made as a reference to evaluate the third component’s effect on the cell efficiency. On average, these cells achieved 11.9% power conversion efficiency, with a short-circuit current density (*J_SC_*) of 24.3 mA/cm^2^ and a fill factor (FF) of 0.60.

**Figure 6:**
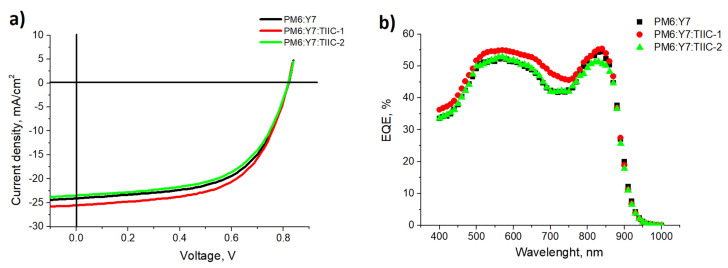
(**a**) Examples of the current-voltage characteristics of the studied solar cells; (**b**) EQE spectral dependence for the studied solar cells.

As the performance of ternary solar cells is susceptible to the concentration of the third component, a series of samples was prepared while varying the amount of **TIIC-1** in the active layer. The lowest amount (sample with a material ratio of **PM6:Y7:TIIC-1** of 10:10:0.5) showed minimal improvement: an increase of around 0.2 mA/cm^2^ in *J_SC_* and 0.1% in PCE, within the margin of error (see Figure S8 and Table S1, Supplementary Materials). The sample with 10:10:1 **PM6:Y7:TIIC-1** showed an increase in the short-circuit current density by almost 1 mA/cm^2^, reaching 25 mA/cm^2^, while FF slightly increased to 0.61 (see Figure 6a and Table 3). This led to an increase in PCE of around 0.6%, bringing it to 12.5%. Further increases in the amount of **TIIC-1** in the active layer lead to decreases in all solar cell parameters (*J_SC_*, *V_OC_*, FF, and PCE), as shown in Figure S8 and Table S1 (Supplementary Materials).

Ternary solar cells containing **TIIC-2** with a 10:10:1 material mass ratio showed slightly lower efficiency than the reference cells. While *V*_OC_ remained unchanged at 0.83 V, the slight decreases in *J_SC_* and FF resulted in a lower PCE of 11.65% for ternary cells with **TIIC-2**, compared to 11.90% for reference cells. While the absorption spectrum of **TIIC-2** lies between those of **PM6** and **Y7** (see Figure 4b), this property alone was not sufficient to improve the results. The reduced efficiency could be due to the deep energy levels of **TIIC-2**. The holes can be easily transported from **TIIC-2** to **Y7** and then to **PM6**, or directly to **PM6**. As the electron affinity level of **TIIC-2** is so close to the work function of the electrode (Ag), some of the electrons generated by **PM6** and **Y7** are transported first to the **TIIC-2,** and from there, to Ag. This extra step can slightly slow electron transport in the solar cell, leading to a slight imbalance in charge-carrier extraction. The evidence of this is the decreased FF in TOSCs containing **TIIC-2**.

Additionally, the EQE spectral dependence (Figure 6b) shows lower TOSC efficiency over the 800–860 nm range. This could be explained by charge transfer from **Y7** to **TIIC-2**, followed by recombination, rather than by charge transfer from **Y7** to the electron transport layer and electrode. Even at the lower wavelengths, the EQE spectral dependence of **TIIC-2** containing ternary cells resembles the EQE spectrum of the reference cells. On the contrary, TOSCs containing **TIIC-1** show increased EQE in the spectral range between 400 nm and 760 nm. In the range between 400 nm and 650 nm, the improvement could be due to increased absorption, while in the range between 650 nm and 760 nm, it is based solely on improved charge-carrier extraction.

Surprisingly, bulk heterojunction cells (cells without **Y7**) containing **TIIC-2** are one order of magnitude more efficient than the cells containing **TIIC-1**. Unfortunately, these efficiencies are only 0.21% and 0.02% for **TIIC-2** and **TIIC-1** cells, respectively (see Figure S9 and Table S2 in Supplementary Materials). This could be related to two main factors. Firstly, the materials themselves could be less efficient than **Y7**. For example, lower charge-carrier mobility can increase recombination losses. Secondly, OSC efficiency heavily depends on the morphology of the active layer. The choice of solvents and the annealing temperature affect phase separation and domain size in the thin film, which, in turn, affect charge transport and extraction in the solar cells.

Additionally, an attempt was made to evaluate the feasibility of using the **TIIC-1** as an electron donor. A solar cell was made with the active layer of **TIIC-1:Y7** (without the polymer **PM6**). In this case, there was absolutely no photovoltaic effect—the measured I–V curve had characteristics of a simple resistor (see Figure S9a, Supplementary Materials).

In this attempt, **TIIC-2** worked better as a solo electron acceptor than **TIIC-1**, but **TIIC-1** was more efficient as a ternary component. While some aspects were less favorable for **TIIC-1** (absorption maximum position, the presence of *E*,*E* and *Z*,*Z* isomers) compared to **TIIC-2**, **TIIC-1** showed better compatibility with the reference system **PM6:Y7**. This shows that a thorough investigation is needed to assess the potential of each novel material. In this work, we do not report devices with record-high performance, yet the addition of a third component (**TIIC-1**) has shown promising improvements in the binary system.

## 4. Conclusions

In this work, we have shown the synthesis and characterization of two Janus-dione-based non-fullerene electron acceptor materials, **TIIC-1** and **TIIC-2**. It was demonstrated that **TIIC-1** is obtained as a mixture of *E*,*E* and *Z*,*Z* isomers at a 1:1 ratio, while **TIIC-2** is obtained as an isomerically pure compound. It could be argued that the presence of two distinct geometrical isomers would be detrimental to the morphological properties and, consequently, to the photovoltaic performance of **TIIC-1**. The observed ten-fold lower PCE of **TIIC-1** as a standalone acceptor compared to **TIIC-2** could rationalize such a claim. Despite this, **TIIC-1** showed markedly higher performance in a ternary blend with **PM6** and **Y7** compared to **TIIC-2**. The **PM6:Y7** reference cell showed a PCE of 11.90%, while the **PM6:Y7:TIIC-1** TOSC reached a PCE of 12.50%. The energy-level measurements showed that **TIIC-1** creates an energy-level cascade between **PM6** and **Y7**, suggesting it can serve as an intermediary for charge-carrier transport or operate in parallel with **Y7**. On the contrary, the deep energy levels of **TIIC-2** and the resulting unfavorable energy level arrangement in the ternary device yield a slightly lower PCE of 11.65%.

## Figures and Tables

**Table 1:** The results of TD-DFT calculations.

Compound	*S*_0_→*S_X_* (eV)	*f*_osc_	*T*_1_ (eV)	*E*_HOMO_ (eV)	*E*_LUMO_ (eV)
**TIIC-1**	2.1 (S_1_)	0.01	1.74	−5.79	−3.23
	2.12 (S_2_)	0			
	2.43 (S_3_)	2.75			
**TIIC-2**	1.79 (S_1_)	0.07	1.53	−5.91	−3.69
	1.79 (S_2_)	0			
	2.04 (S_3_)	1.14			

**Table 2:** Electrochemical data for compounds **TIIC-1** and **TIIC-2**. Peak onset potentials were used to determine the oxidation and reduction potentials. The HOMO energy level was calculated as *E*_HOMO_ = −(*E*_ox_ + 5.1) (eV), while the LUMO energy level was estimated as *E*_LUMO_= −(*E*_red_ + 5.1) (eV) [41].

Compound	*E*_onset ox_ (V)	*E*_onset red_ (V)	Δ*E* (V)	*E*_HOMO_ (eV)	*E*_LUMO_ (eV)
**TIIC-1**	0.54	−1.26	1.80	−5.64	−3.84
**TIIC-2**	0.65	−0.82	1.47	−5.75	−4.28

**Table 3:** Summary of the solar cell parameters.

Active Layer	*J*_SC_, mA/cm^2^	*V*_OC_, V	FF	PCE, %
**PM6:Y7**	24.3	0.83	0.60	11.90
**PM6:Y7:TIIC-1**	25.1	0.82	0.61	12.50
**PM6:Y7:TIIC-2**	23.9	0.83	0.59	11.65

## Data Availability

The original contributions presented in this study are included in the article/Supplementary Materials. Further inquiries can be directed to the corresponding author.

## Supplementary Materials

The following supporting information can be downloaded at: https://www.mdpi.com/article/10.3390/ma19030533/s1, Scheme S1. Synthesis of compounds **TIIC-1** and **TIIC-2**. Figure S1. ^1^H NMR spectrum of **TIIC-1**, aromatic proton signals (CDCl_3_, 500 MHz, room temperature). Figure S2. ^1^H NMR spectra of **TIIC-1**, aromatic proton signals (C_2_Cl_4_D_2_, 500 MHz). (a) At room temperature (298 K). (b) At 70 °C (343 K). Figure S3. Structures of possible rotational isomers for **TIIC-1**. Figure S4. The natural transition orbitals (NTOs) for **TIIC-1**. Determined at PBE0/def2-TZVP level. Figure S5. The natural transition orbitals (NTOs) for **TIIC-2**. Determined at PBE0/def2-TZVP level. Figure S6. Cyclic voltammograms of compounds **TIIC-1** (a) and **TIIC-2** (b). Measured in CH_2_Cl_2_; supporting electrolyte—TBAF (0.1 M); scan rate of 50 mV/s; working electrode—glassy carbon disk; counter electrode—Pt wire; reference electrode—Ag/Ag^+^ (0.1 M). The potentials were calibrated against Fc/Fc^+^ redox couple. Figure S7. PL quenching experiment between **TIIC-1** and **Y7** in toluene solution. An excitation wavelength of 570 nm was used. Figure S8. Current-voltage characteristics of ternary solar cells depending on the **PM6:Y7:TIIC-1** mass ratio. Table S1. Summary of solar cell parameters depending on PM6:Y7:TIIC-1 mass ratio. Figure S9. Current-voltage characteristics of bulk heterojunction solar cells with (a) **PM6:TIIC-1** and **TIIC-1:Y7**, and (b) **PM6:TIIC-2** as the active layer materials. Table S2. Summary of binary solar cell parameters. Figures S10–S23. ^13^C NMR spectra.

## Abbreviations

The following abbreviations are used in this manuscript:

A	Acceptor
BHJ	Bulk heterojunction
CT	Charge transfer
CV	Cyclic voltammetry
D	Donor
DFT	Density functional theory
EQE	External quantum efficiency
ESP	Electrostatic potential
FF	Fill factor
HOMO	Highest occupied molecular orbital
ITO	Indium tin oxide
LUMO	Lowest unoccupied molecular orbital
NFA	Non-fullerene acceptor
NMR	Nuclear magnetic resonance
NTO	Natural transition orbital
OSC	Organic solar cell
PCE	Power conversion efficiency
PCM	Polarizable continuum model
PYS	Photoemission yield spectroscopy
TD-DFT	Time-dependent density functional theory
TOSC	Ternary organic solar cell
