# Supplementary material for: Synthesis and Study of Janus-Dione-Based Compounds for Ternary Organic Solar Cells
Source: Materials (Basel). 2026 Jan 29;19(3):533. doi: 10.3390/ma19030533 (PMC12898524; doi:10.3390/ma19030533)
Supplement: Supplementary file 1 [file materials-19-00533-s001.zip › materials-4077633-supplementary.pdf]

Supporting Materials for

Synthesis and study of Janus-dione-based compounds for ternary organic solar cells

Armands Ruduss[1], Anastasija Rizkova[1], Fatima Zohra Boudjenane[1], Elizabete Praulina[2], Kaspars Traskovskis[1], Raitis Grzibovskis[2]

[1]Riga Technical University, Faculty of Materials Science and Applied Chemistry, 3/7 Paula Valdena Street, Riga LV-1048, Latvia.

[2]Institute of Solid State Physics, University of Latvia, 8 Kengaraga street, Riga, LV-1063, Latvia

Synthesis

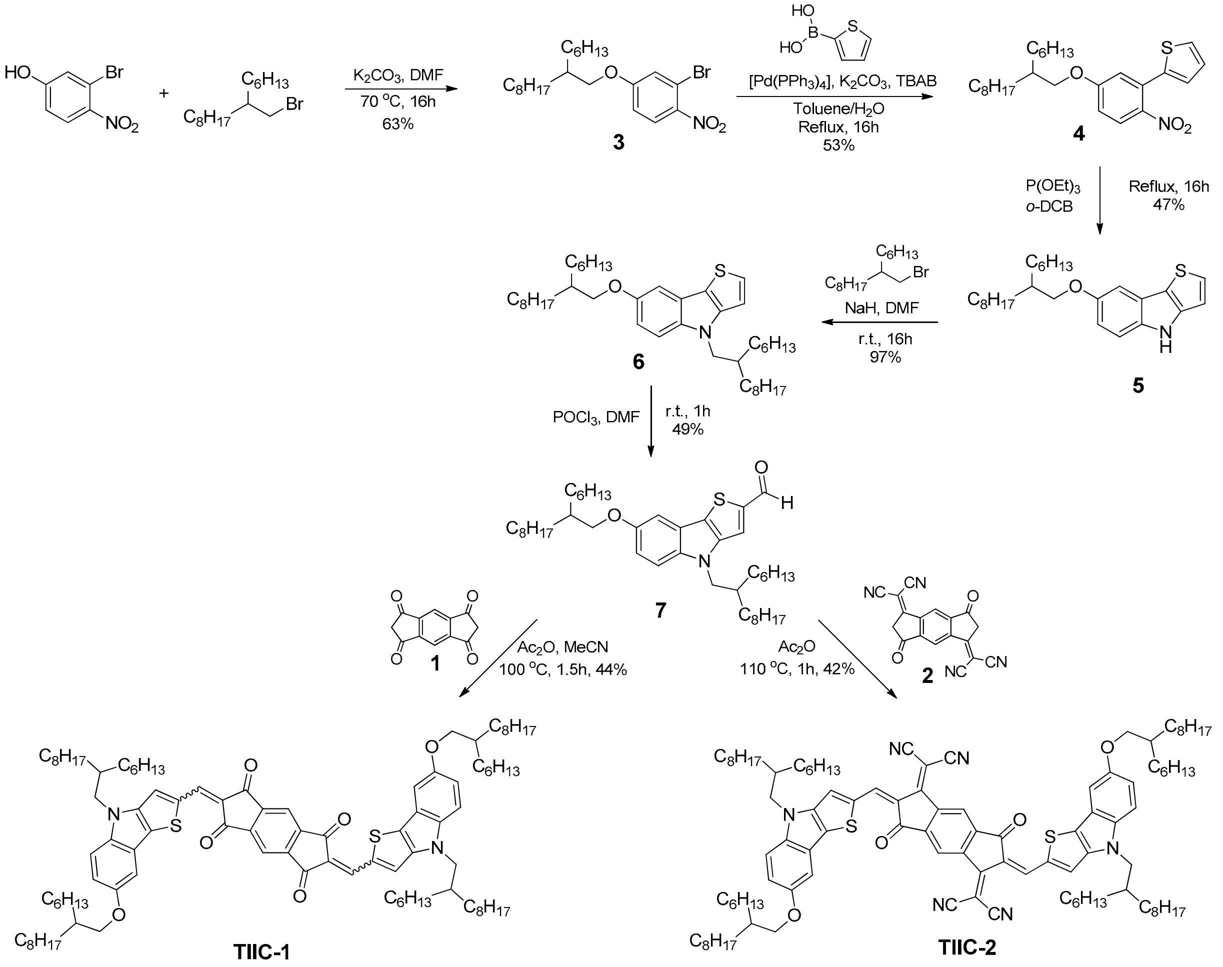

Scheme S1. Synthesis of compounds **TIIC-1** and **TIIC-2.**

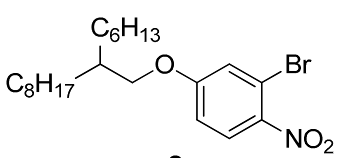

3

Compound 3, 2-bromo-4-((2-hexyldecyl)oxy)-1-nitrobenzene.

3-Bromo-4-nitrophenol (1.50 g, 6.88 mmol) was dissolved in DMF (30 ml), and K_2CO_3 (1.91 g, 13.82 mmol) was added, and the reaction was left to stir for 30 min at room temperature. Then the 7-(bromomethyl)pentadecane (3.18 g, 10,41 mmol) was added and the reaction was heated at 70 °C with stirring overnight (16 h). Then the reaction mixture was cooled to room temperature and water was added. The resulting mixture was extracted with DCM, washed with saturated NaCl solution, dried over sodium sulfate, and evaporated under reduced pressure. The crude product was purified by flash column chromatography (silica gel, EtOAc : Hex = 1 : 50) to obtain a yellow oil (1.92 g, 63%).

^{1}H NMR δH ($CDCl_3$, 500 MHz): 7.98 (d, J = 9.1 Hz, 1H), 7.21 (d, J = 1.6 Hz, 1H), 6.90 (dd, J = 9.1, 1.6 Hz, 1H), 3.89 (d, J = 5.5 Hz, 2H), 1.79 (hept, J = 5.8 Hz, 1H), 1.44 – 1.22 (m, 24H), 0.90 – 0.85 (m, 6H).

^{13}C NMR δC ($CDCl_3$, 125.77 MHz): 162.78, 142.26, 128.14, 120.70, 116.97, 114.00, 72.12, 37.89, 32.03, 31.95, 31.31, 31.29, 30.07, 29.74, 29.69, 29.44, 26.91, 26.89, 22.81, 22.80, 14.26, 14.24.

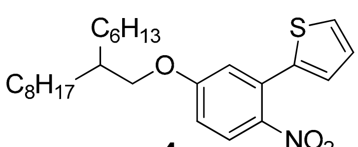

4

Compound 4, 2-(5-((2-hexyldecyl)oxy)-2-nitrophenyl)thiophene.

Under an argon atmosphere, compound **3** (1.85 g, 4.18 mmol), thiophen-2-ylboronic acid (0.70 g, 5.47 mmol) and TBAB (0.17 g, 0.53 mmol) were dissolved in 15 mL of toluene and 10 mL of 2M K_2CO_3. The mixture was degassed by bubbling argon through the mixture for 1 h. Then [Pd(PPh_3)$_4$] (0.24 g, 0.21 mmol) was added and argon was bubbled through the mixture for another 30 min. Then the reaction was heated at reflux for 16 h. Then the reaction mixture was cooled to room temperature and water was added. The product was extracted with DCM, washed with saturated NaCl solution, dried over sodium sulfate, and evaporated under reduced pressure. The obtained crude product was purified by flash column chromatography (silica gel, DCM : Hex = 1 : 5) to obtain a brown oil (1.00 g, 53%).

^{1}H NMR δH ($CDCl_3$, 500 MHz): 7.89 (d, J = 9.0 Hz, 1H), 7.41 (t, J = 3.3 Hz, 1H), 7.09 – 7.06 (m, 2H), 6.96 (d, J = 2.7 Hz, 1H), 6.92 (dd, J = 9.0, 2.7 Hz, 1H), 3.91 (d, J = 5.6 Hz, 2H), 1.80 (hept, J = 6.1 Hz, 1H), 1.47 – 1.22 (m, 24H), 0.92 – 0.85 (m, 6H).

^{13}C NMR δC ($CDCl_3$, 125.77 MHz): 162.05, 142.027, 138.17, 131.54, 127.53, 127.18, 126.96, 126.82, 118.13, 114.05, 71.78, 37.92, 32.00, 31.93, 31.34, 31.33, 30.06, 29.74, 29.67, 29.42, 26.91, 26.89, 22.79, 22.78, 14.22.

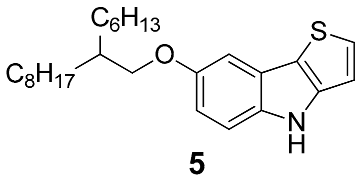

5

Compound 5, 7-((2-Hexyldecyl)oxy)-4*H*-thieno[3,2-*b*]indole.

Compound **4** (0.96 g, 2.15 mmol) was dissolved in dry *o*-DCB (45 ml), and P(OEt)$_3$ (0.89 g, 5.36 mmol) was added, after which the reaction mixture was heated with stirring at reflux for 16 h. After cooling to room temperature, *o*-DCB was removed under reduced pressure and the crude product was purified by flash column chromatography (silica gel, DCM : Hex = 1 : 3) to obtain a yellow oil (0.42 g, 47%).

^{1}H NMR δH ($CDCl_3$, 500 MHz): 8.00 (s, 1H), 7.35 – 7.27 (m, 2H), 7.22 (s, 1H), 7.03 (d, J = 5.1 Hz, 1H), 6.93 (d, J = 8.7, 1H), 3.91 (d, J = 5.6 Hz, 2H), 1.83 (hept, J = 5.6 Hz, 1H), 1.55 – 1.22 (m, 24H), 0.93 – 0.86 (m, 6H).

^{13}C NMR δC ($CDCl_3$, 125.77 MHz): 153.90, 143.79, 136.06, 126.78, 122.51, 117.79, 113.13, 112.50, 111.62, 102.36, 71.90, 38.16, 31.95, 31.92, 31.50, 31.49, 30.11, 29.78, 29.65, 29.39, 26.91, 26.89, 22.73, 14.16.

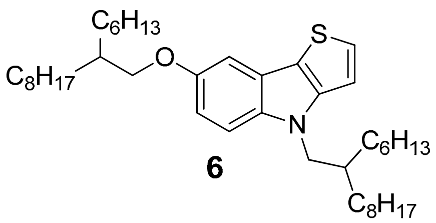

6

*Compound **6***, 4-(2-hexyldecyl)-7-((2-hexyldecyl)oxy)-4*H*-thieno[3,2-*b*]indole.

To a solution of compound **5** (0.24 g, 0.58 mmol) in dry DMF (4 ml), NaH (60 wt% dispersion in mineral oil) (0.035 g, 0.88 mmol) was added at 0 °C. After the addition reaction, the mixture was stirred at room temperature for 90 min. Then 7-(bromomethyl)pentadecane (0.23 g, 0.75 mmol) was added and the mixture was stirred overnight (16 h) at room temperature. The reaction mixture was quenched with water, extracted with EtOAc, washed with saturated NaCl solution, dried over sodium sulfate, and evaporated under reduced pressure. The crude product was purified by flash column chromatography (silica gel, hexane) to obtain the product as a brown oil (0.36 g, 97%).

^{1}H NMR δH ($CDCl_3$, 500 MHz): 7.35 (d, J = 5.2 Hz, 1H), 7.29 – 7.27 (m, 1H), 7.24 – 7.22 (m, 1H), 7.03 (d, J = 5.2 Hz, 1H), 6.96 (dd, J = 8.9, 2.2 Hz, 1H), 4.09 (d, J = 7.3 Hz, 2H), 3.93 (d, J = 5.7 Hz, 2H), 2.06 (hept, J = 5.5 Hz, 1H), 1.85 (hept, J = 5.5 Hz, 1H), 1.47 – 1.18 (m, 48H), 0.94 – 0.86 (m, 12H).

^{13}C NMR δC ($CDCl_3$, 125.77 MHz): 153.43, 146.36, 136.88, 126.48, 121.94, 115.31, 112.72, 110.80, 110.79, 102.44, 72.01, 49.95, 38.73, 38.30, 32.07, 32.05, 32.01, 31.93, 31.87, 31.62, 30.24, 30.05, 29.90, 29.78, 29.74, 29.65, 29.51, 29.40, 27.04, 27.01, 26.62, 22.85, 22.84, 22.81, 22.75, 14.28, 14.23.

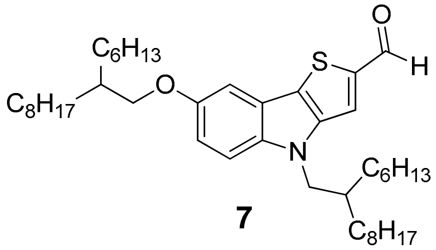

7

Compound 7, 4-(2-hexyldecyl)-7-((2-hexyldecyl)oxy)-4*H*-thieno[3,2-*b*]indole-2-carbaldehyde.

To a dry DMF (0.3 ml, 3.96 mmol), $POCl_3$ (0.13 ml, 1.38 mmol) was slowly added under an argon atmosphere at 0 °C. The resulting mixture was stirred at 0 °C for 45 min. Then a solution of **6** (0.55 g, 0.86 mmol) in dry DMF (3 ml) was slowly added to the formylating reagent, and the reaction mixture was stirred at 50 °C for 2 h. Then the reaction mixture was cooled to room temperature and quenched with water. The mixture was extracted with EtOAc, washed with saturated NaCl solution, dried over sodium sulfate, and evaporated under reduced pressure. The crude product was purified by flash column chromatography (silica gel, DCM : Hex = 1 : 1.5) to obtain a yellow oil (0.28 g, 49%).

^{1}H NMR δH ($CDCl_3$, 500 MHz): 9.92 (s, 1H), 7.63 (s, 1H), 7.25 – 7.21 (m, 2H), 7.04 (d, J = 8.9 Hz, 1H), 4.07 (d, J = 7.4 Hz, 2H), 3.89 (d, J = 5.5 Hz, 2H), 2.01 (hept, J = 5.1 Hz, 1H), 1.80 (hept, J = 5.2 Hz, 1H), 1.40 – 1.14 (m, 48H), 0.89 – 0.80 (m, 12H).

^{13}C NMR δC ($CDCl_3$, 125.77 MHz): 183.52, 153.96, 145.02, 143.25, 138.85, 123.97, 121.11, 118.77, 116.81, 111.43, 102.97, 71.86, 50.02, 38.72, 38.26, 32.06, 32.03, 31.98, 31.88, 31.60, 31.59, 30.20, 30.01, 29.87, 29.76, 29.70, 29.60, 29.49, 29.37, 27.03, 27.01, 26.60, 26.59, 22.83, 22.78, 22.73, 14.27, 14.25, 14.21.

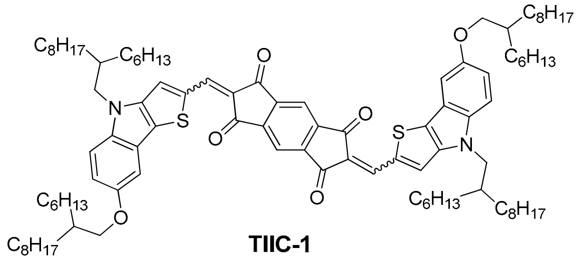

TIIC-1

*Compound **TIIC-1***. 2,6-bis((4-(2-hexyldecyl)-7-((2-hexyldecyl)oxy)-4*H*-thieno[3,2-*b*]indol-2-yl)methylene)-*s*-indacene-1,3,5,7(2*H*,6*H*)-tetraone.

Compound **7** (0.24 g, 0.36 mmol) and *s*-indacene-1,3,5,7(2*H*,6*H*)-tetraone (**8**) (0.07 g, 0.33 mmol) were dissolved in Ac_2O (10 ml), and the reaction mixture was heated with stirring at 110 °C for 2 h. Then the reaction mixture was cooled to room temperature and the precipitated dark solid containing the crude product was separated by filtration and washed with MeOH. The crude product was purified by flash column chromatography (silica gel, DCM : Hex = 1 : 1) to obtain a dark solid (0.12 g, 44%).

^{1}H NMR *δ*H ($CDCl_3$, 500 MHz): 8.12 – 8.02 (m, 2H), 7.87 – 7.68 (m, 2H), 7.14 – 7.07 (m, 4H), 6.97 – 6.92 (m, 2H), 4.10 – 3.97 (m, 4H), 3.88 – 3.78 (m, 4H), 2.04 – 1.93 (m, 2H), 1.84 – 1.75 (m, 2H), 1.47 – 1.14 (m, 96H), 0.94 – 0.80 (m, 12H).

^{13}C NMR *δ*C ($CDCl_3$, 125.77 MHz): 188.77, 188.68, 187.28, 187.15, 154.08, 146.44, 146.20, 144.55, 144.32, 139.93, 138.62, 120.99, 118.70, 116.41, 116.32, 116.18, 111.56, 102.91, 71.88, 49.94, 38.69, 38.18, 32.10, 32.08, 32.01, 31.91, 31.52, 30.30, 30.08, 29.96, 29.83, 29.76, 29.63, 29.56, 29.43, 27.02, 26.99, 26.65, 22.89, 22.86, 22.80, 22.77, 14.31, 14.30, 14.25.

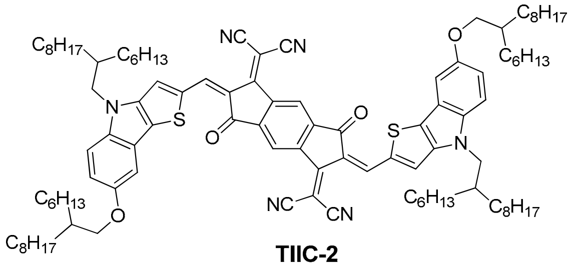

TIIC-2

*Compound **TIIC-2***. 2,2'-((2*Z*,6*Z*)-2,6-bis((4-(2-hexyldecyl)-7-((2-hexyldecyl)oxy)-4*H*-thieno[3,2-*b*]indol-2-yl)methylene)-3,7-dioxo-2,3,6,7-tetrahydro-*s*-indacene-1,5-diylidene)dimalononitrile

Compound **7** (0.27 g, 0.41 mmol) and 2,2'-(3,7-dioxo-2,3,6,7-tetrahydro-*s*-indacene-1,5-diylidene)dimalononitrile (**9**) (0.12 g, 0.39 mmol) were dissolved in dry MeCN (10 ml). A catalytic amount of Ac_2O (0.2 ml) was added and the reaction mixture was refluxed with stirring for 1.5 h. Then the reaction mixture was cooled to room temperature and the precipitated dark solid containing the crude product was separated by filtration and washed with MeOH. Crude product was purified by flash column chromatography (silica gel, DCM : Hex = 1 : 1) to obtain a dark solid (0.14 g, 42%).

^{1}H NMR *δ*H ($CDCl_3$, 500 MHz): 8.92 – 8.81 (m, 4H), 7.92 – 7.59 (m, 2H), 7.24 – 7.20 (m, 2H), 7.16 – 7.08 (m, 4H), 4.04 – 3.96 (m, 4H), 3.96 – 3.89 (m, 4H), 1.99 – 1.92 (m, 2H), 1.89 – 1.81 (m, 2H), 1.40 – 1.13 (m, 96H), 0.93 – 0.79 (m, 12H).

^{13}C NMR δC ($CDCl_3$, 125.77 MHz): 185.57, 157.48, 154.38, 146.42, 143.35, 140.94, 140.88, 139.41, 139.39, 138.74, 135.56, 121.29, 120.99, 120.50, 119.09, 114.65, 114.39, 111.72, 103.03, 71.86, 69.87, 49.98, 38.40, 38.07, 31.96, 31.93, 31.84, 31.70, 31.67, 31.39, 30.14, 29.89 29.80, 29.71, 29.68, 29.58, 29.44, 29.41, 29.25, 26.90, 26.87, 26.44, 26.41, 22.74, 22.73, 22.65, 22.60, 14.17, 14.12, 14.06.

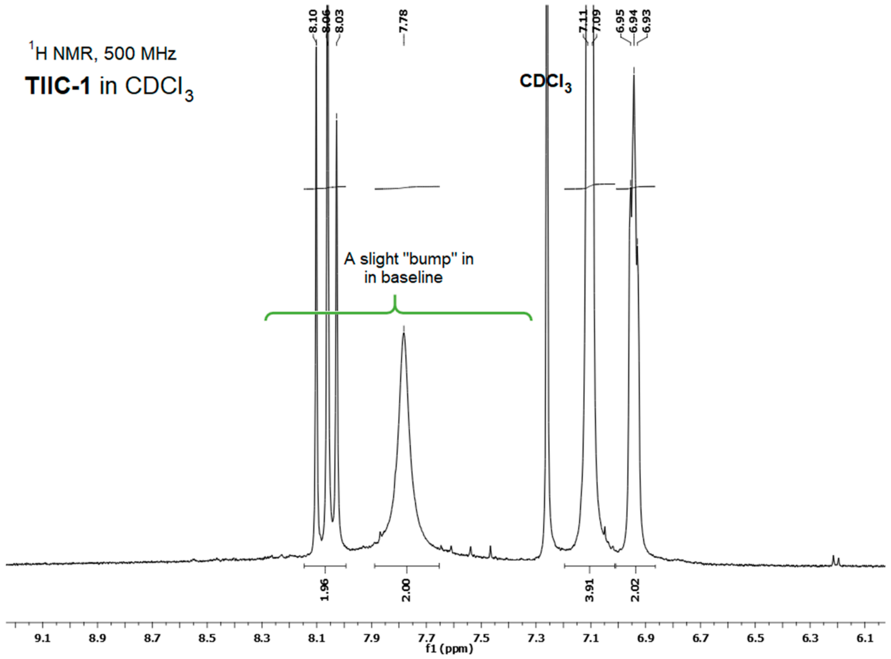

Figure S1. ^1H NMR spectrum of **TIIC-1**, aromatic proton signals ($CDCl_3$, 500 MHz, room temperature).

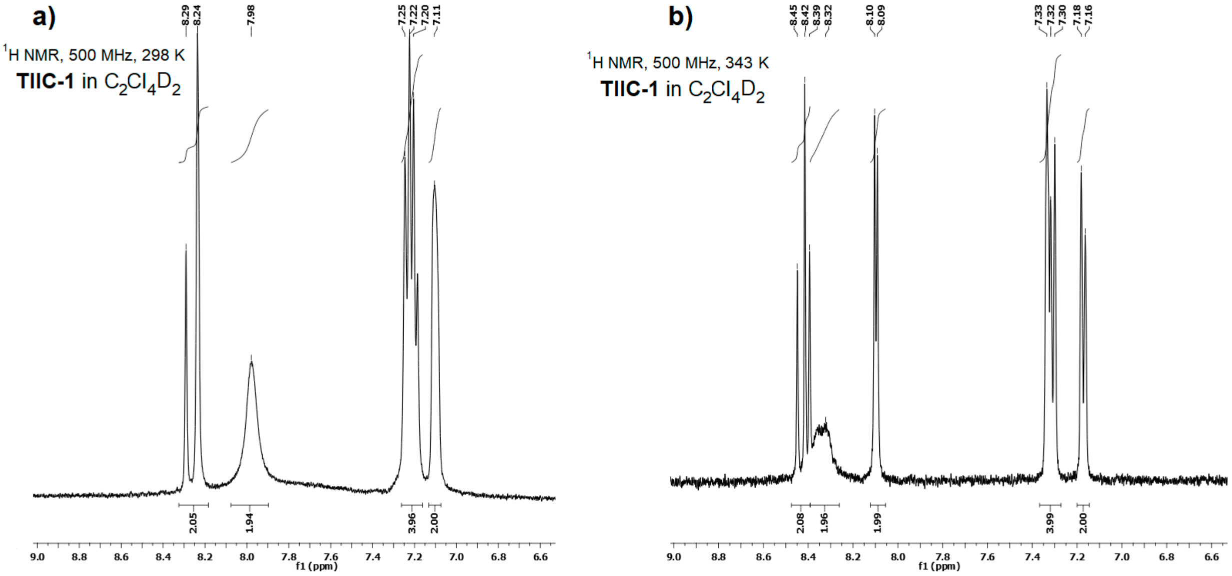

Figure S2. ^1H NMR spectra of **TIIC-1**, aromatic proton signals ($C_2Cl_4D_2$, 500 MHz). (a) At room temperature (298 K). (b) At 70 °C (343 K).

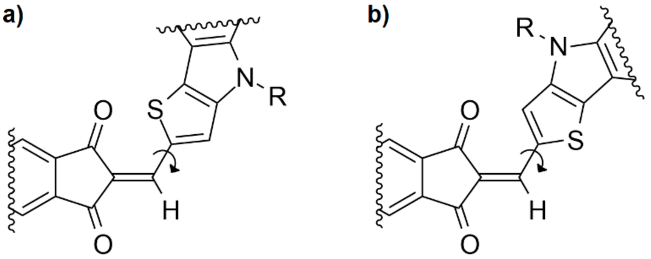

Figure S3. Structures of possible rotational isomers for **TIIC-1**.

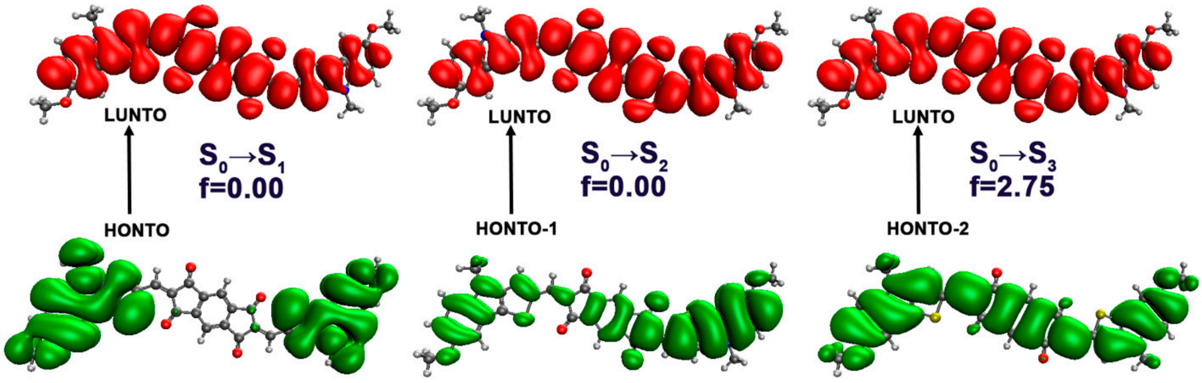

Figure S4. The natural transition orbitals (NTOs) for **TIIC-1**. Green NTOs correspond to a hole and red to a particle. Determined at PBE0/def2-TZVP level.

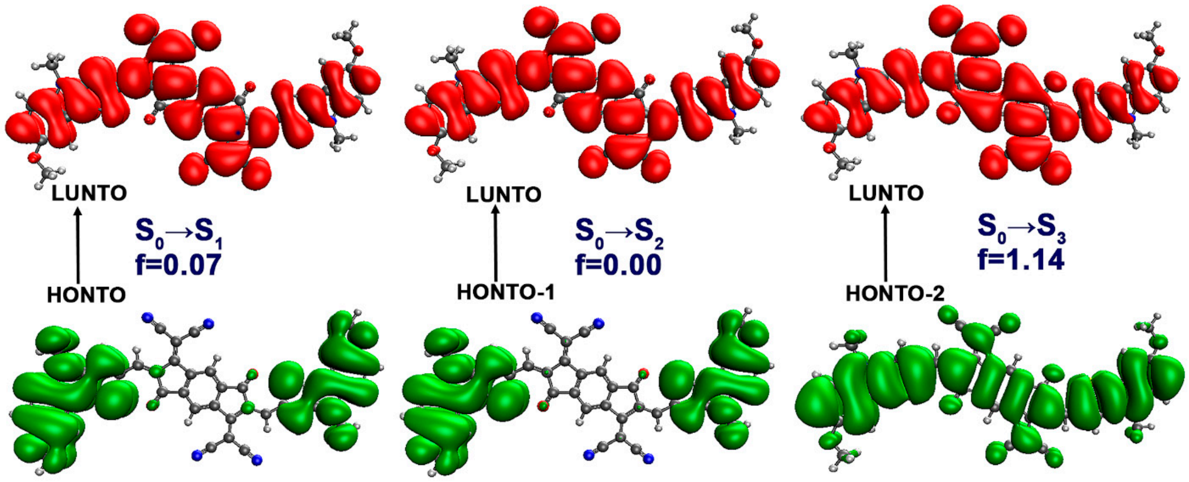

Figure S5. The natural transition orbitals (NTOs) for **TIIC-2**. Green NTOs correspond to a hole and red to a particle. Determined at PBE0/def2-TZVP level.

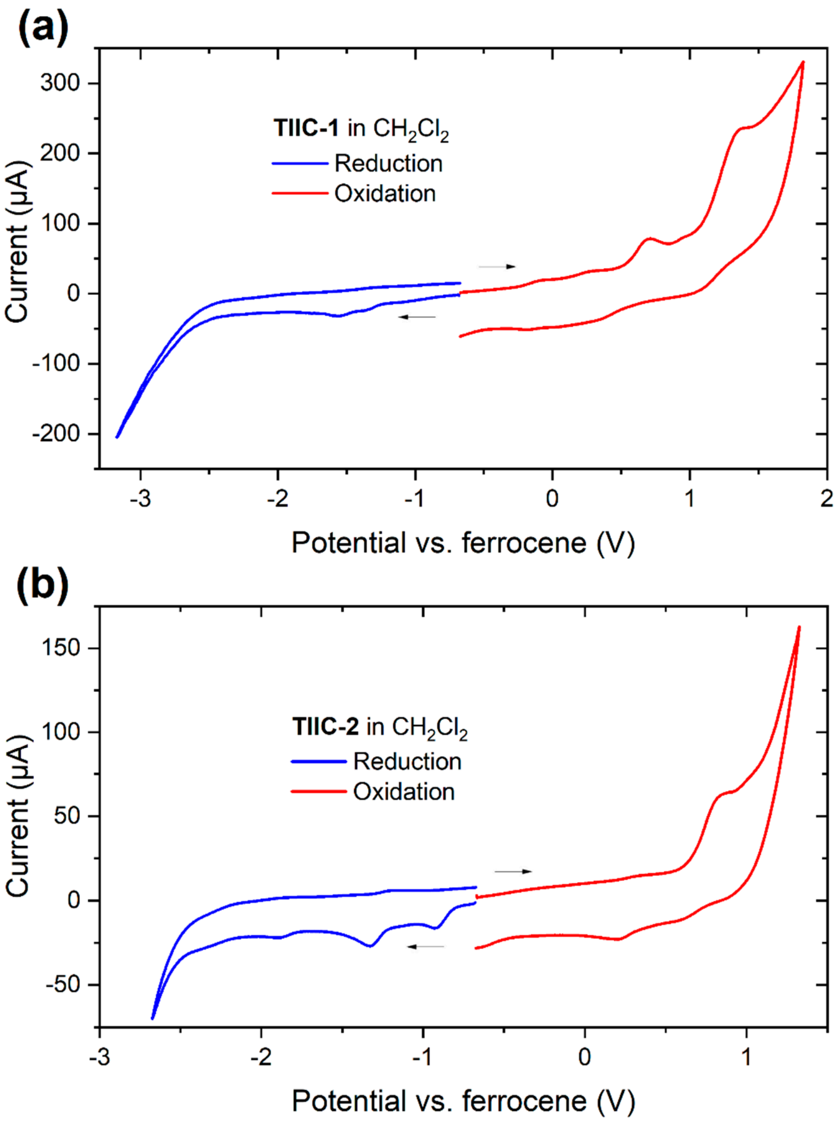

Figure S6. Cyclic voltammograms of compounds **TIIC-1** (a) and **TIIC-2** (b). Measured in CH_2Cl_2; supporting electrolyte—TBAF (0.1 M); scan rate of 50 mV/s; working electrode—glassy carbon disk; counter electrode—Pt wire; reference electrode—Ag/Ag^+ (0.1M). The potentials were calibrated against a Fc/Fc^+ redox couple.

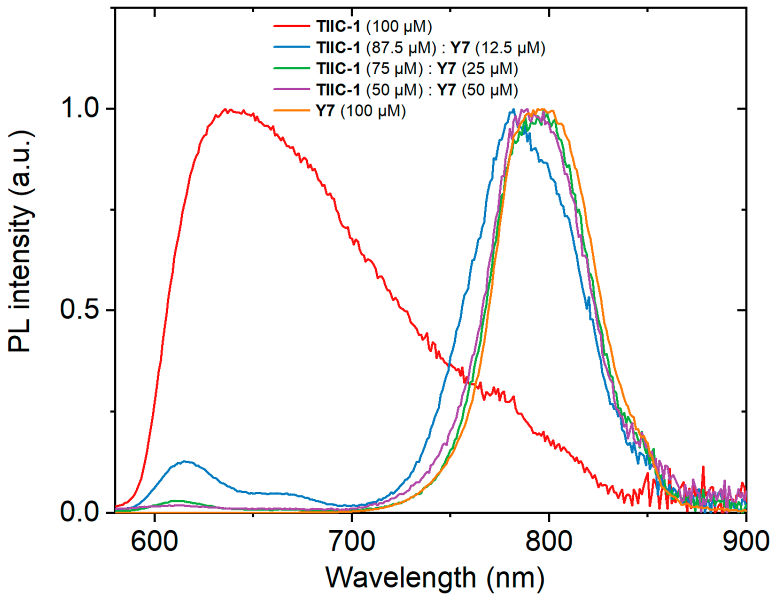

Figure S7. PL quenching experiment between **TIIC-1** and **Y7** in toluene solution. An excitation wavelength of 570 nm was used.

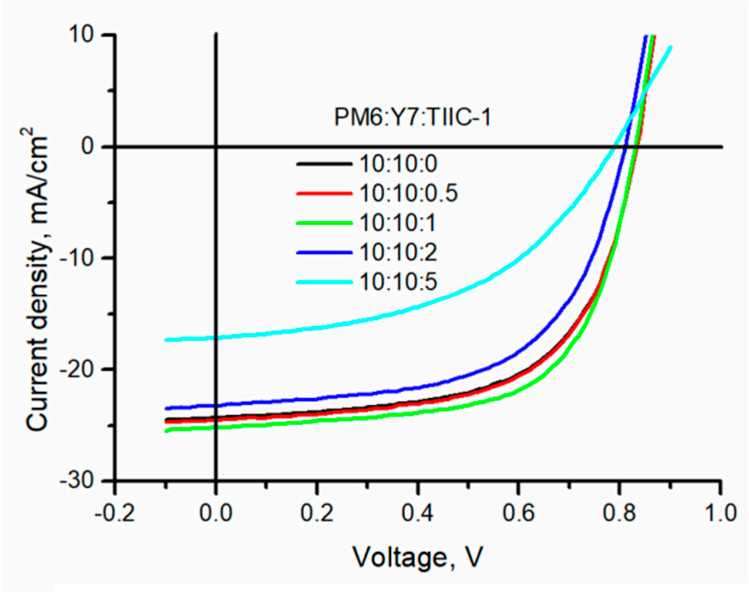

Figure S8. Current–voltage characteristics of ternary solar cells depending on the **PM6**:**Y7**:**TIIC-1** mass ratio.

Table S1. Summary of solar cell parameters depending on PM6:Y7:TIIC-1 mass ratio.

PM6:Y7:TIIC-1 mass ratio	J_{SC}, mA/cm²	V_{OC}, V	FF	PCE, %
**10:10:0**	24.3	0.83	0.60	11.9
**10:10:0.5**	24.5	0.82	0.60	12.0
**10:10:1**	25.1	0.82	0.61	12.5
**10:10:2**	23.2	0.81	0.59	11.1
**10:10:5**	17.1	0.78	0.48	6.4
	±0.2	±0.01	±0.01	±0.2

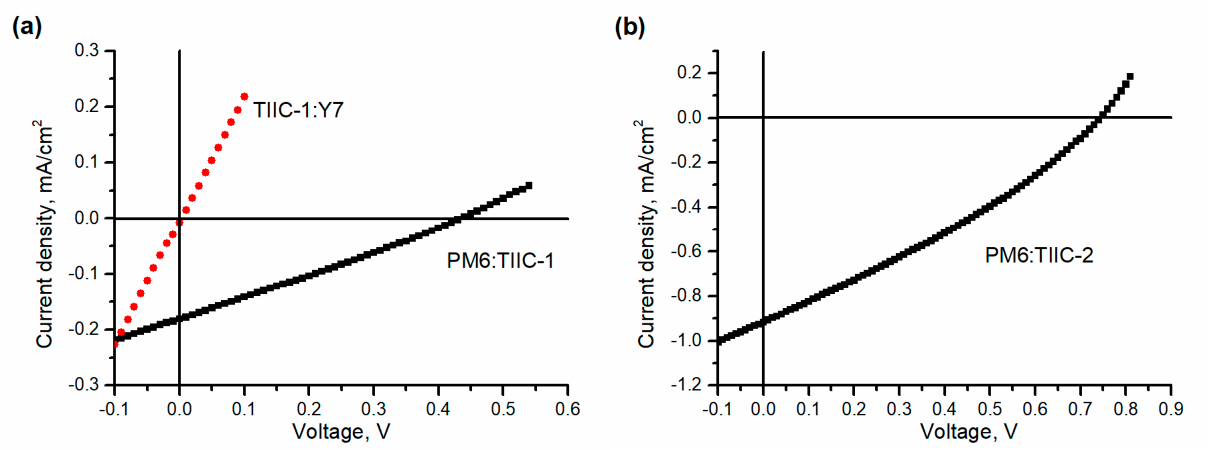

Figure S9. Current–voltage characteristics of bulk heterojunction solar cells with **(a) PM6**:**TIIC-1** and **TIIC-1:Y7**, and **(b) PM6**:**TIIC-2**, as the active layer materials.

Table S2. Summary of binary solar cell parameters.

Active layer	J_{SC}, mA/cm²	V_{OC}, V	FF	PCE, %
**PM6**:**Y7**	24.3	0.83	0.60	11.90
**PM6**:**TIIC-1**	0.20	0.44	0.26	0.02
**PM6**:**TIIC-2**	1.01	0.75	0.30	0.21

NMR Spectra

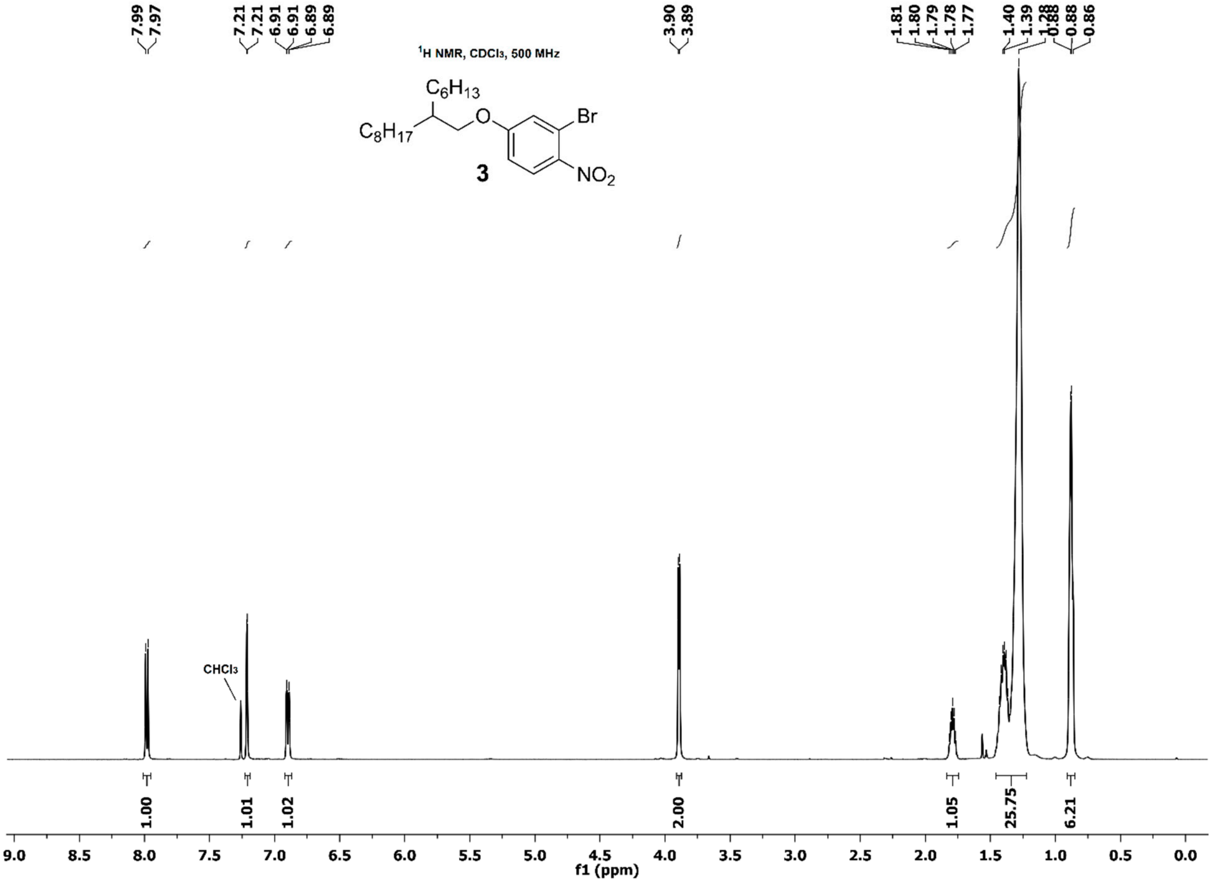

Figure S10. ^{1}H NMR spectrum of **3** ($CDCl_3$, 500 MHz).

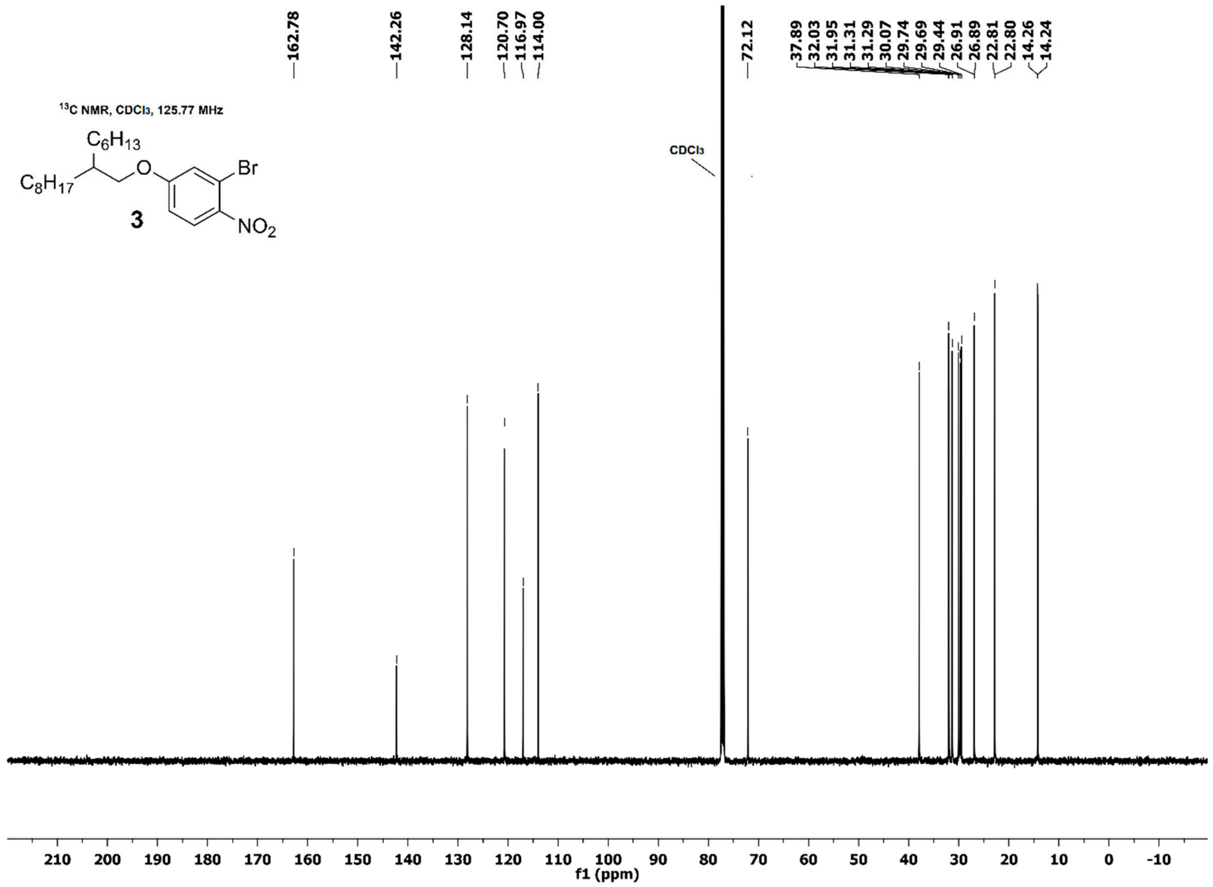

Figure S11. ^{13}C NMR spectrum of **3** ($CDCl_3$, 125.77 MHz).

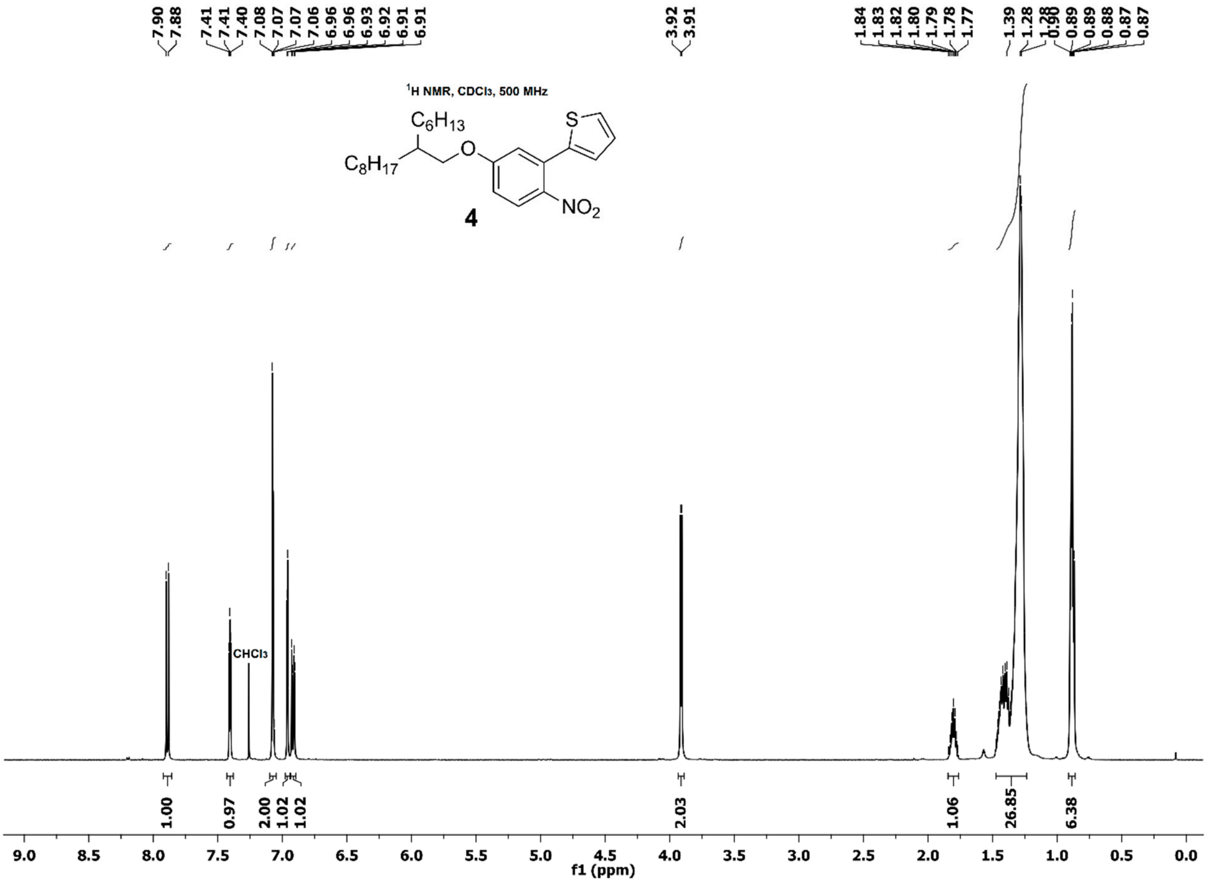

Figure S12. ^{1}H NMR spectrum of **4** ($CDCl_3$, 500 MHz).

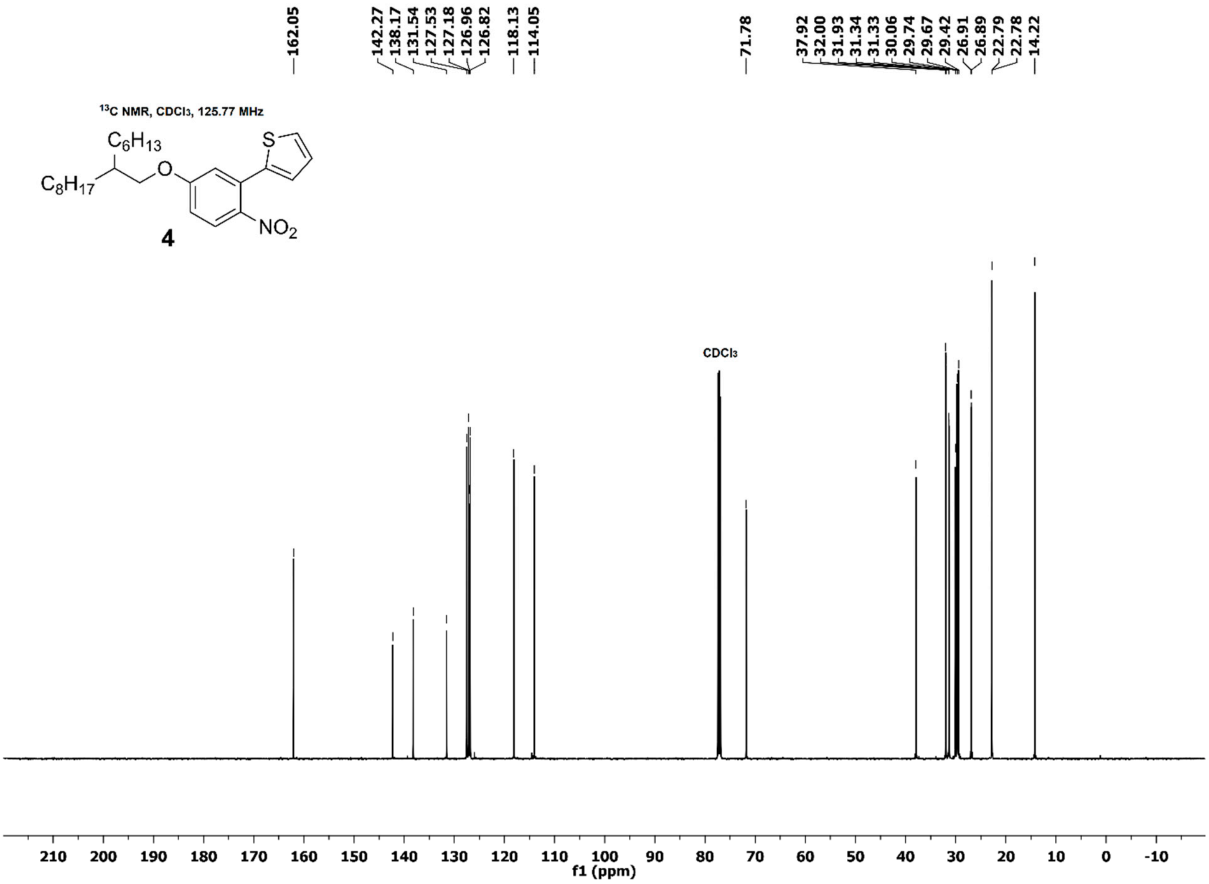

Figure S13. ^{13}C NMR spectrum of **4** ($CDCl_3$, 125.77 MHz).

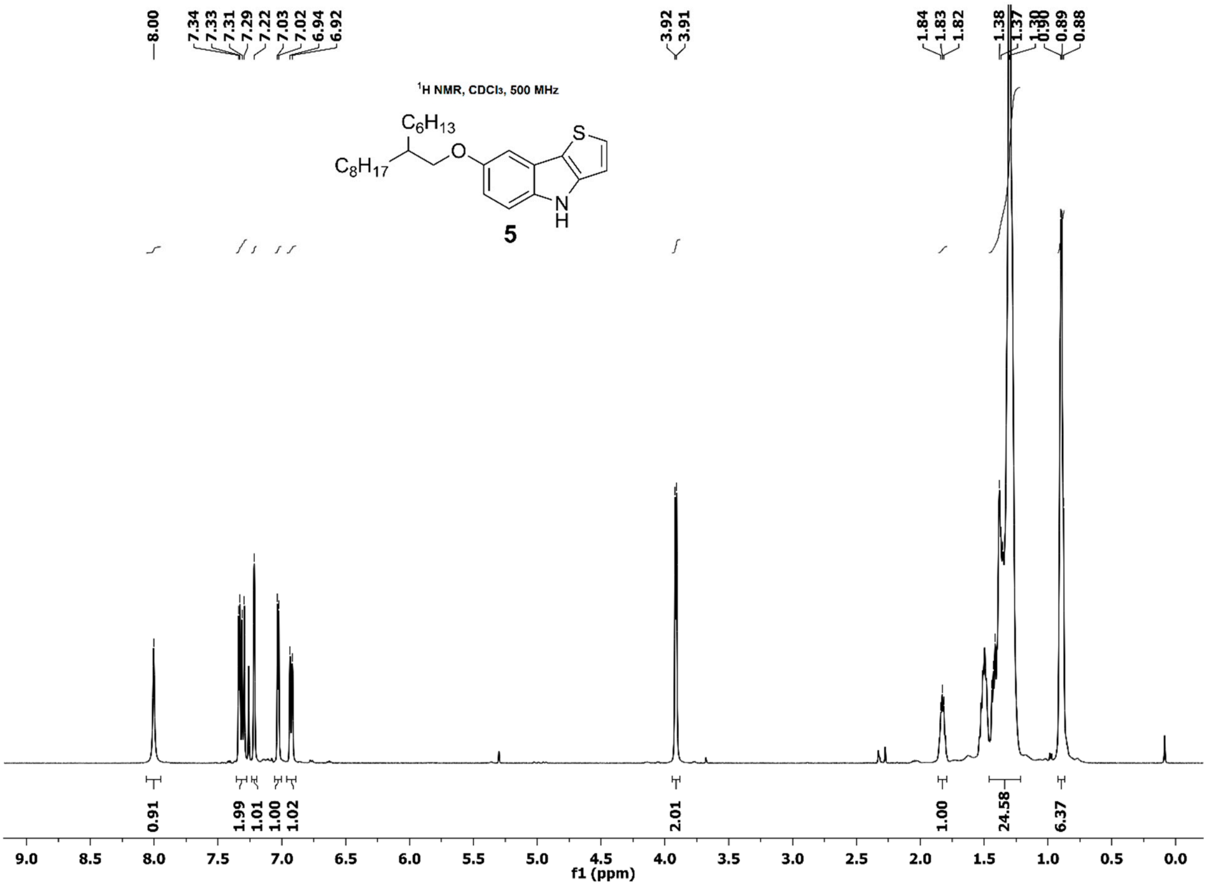

Figure S14. ^{1}H NMR spectrum of **5** ($CDCl_3$, 500 MHz).

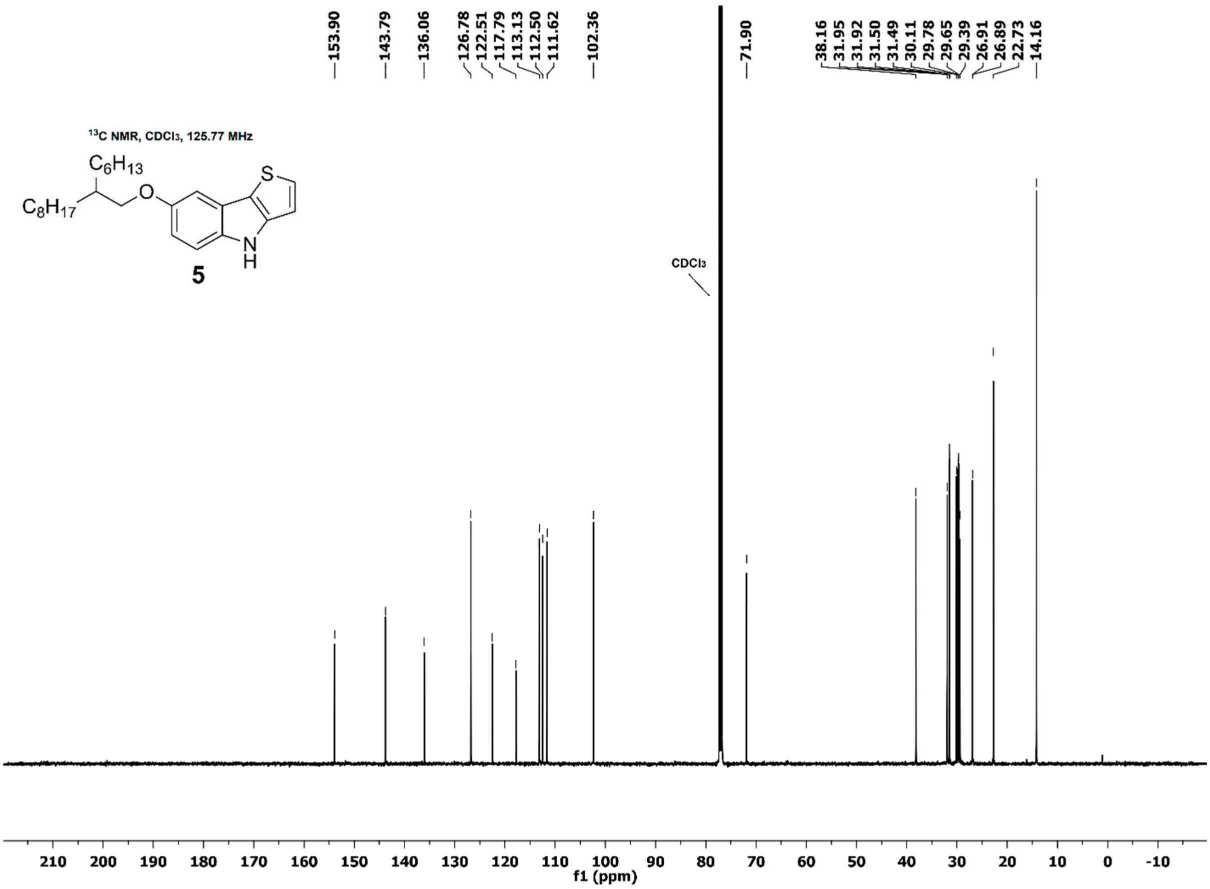

Figure S15. ^{13}C NMR spectrum of **5** ($CDCl_3$, 125.77 MHz).

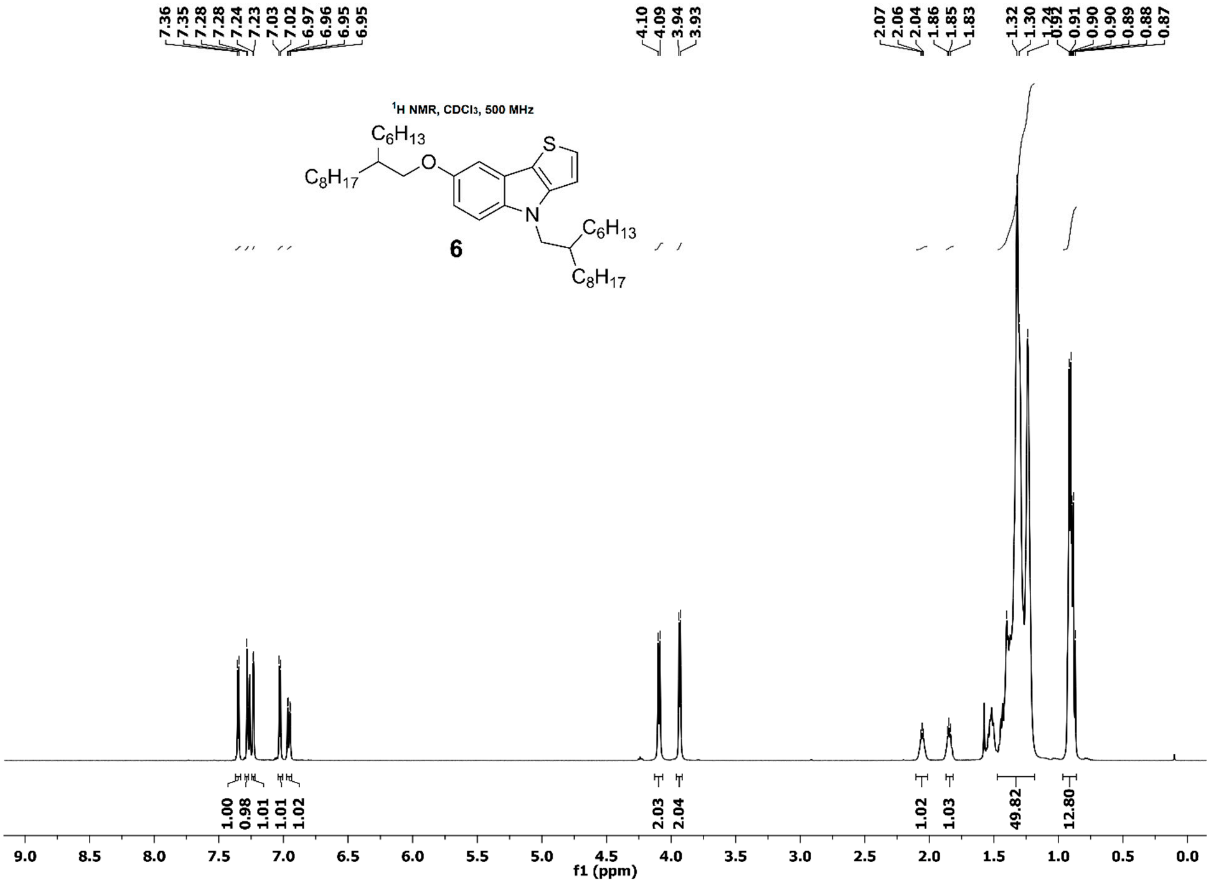

Figure S16. ^{1}H NMR spectrum of **6** ($CDCl_3$, 500 MHz).

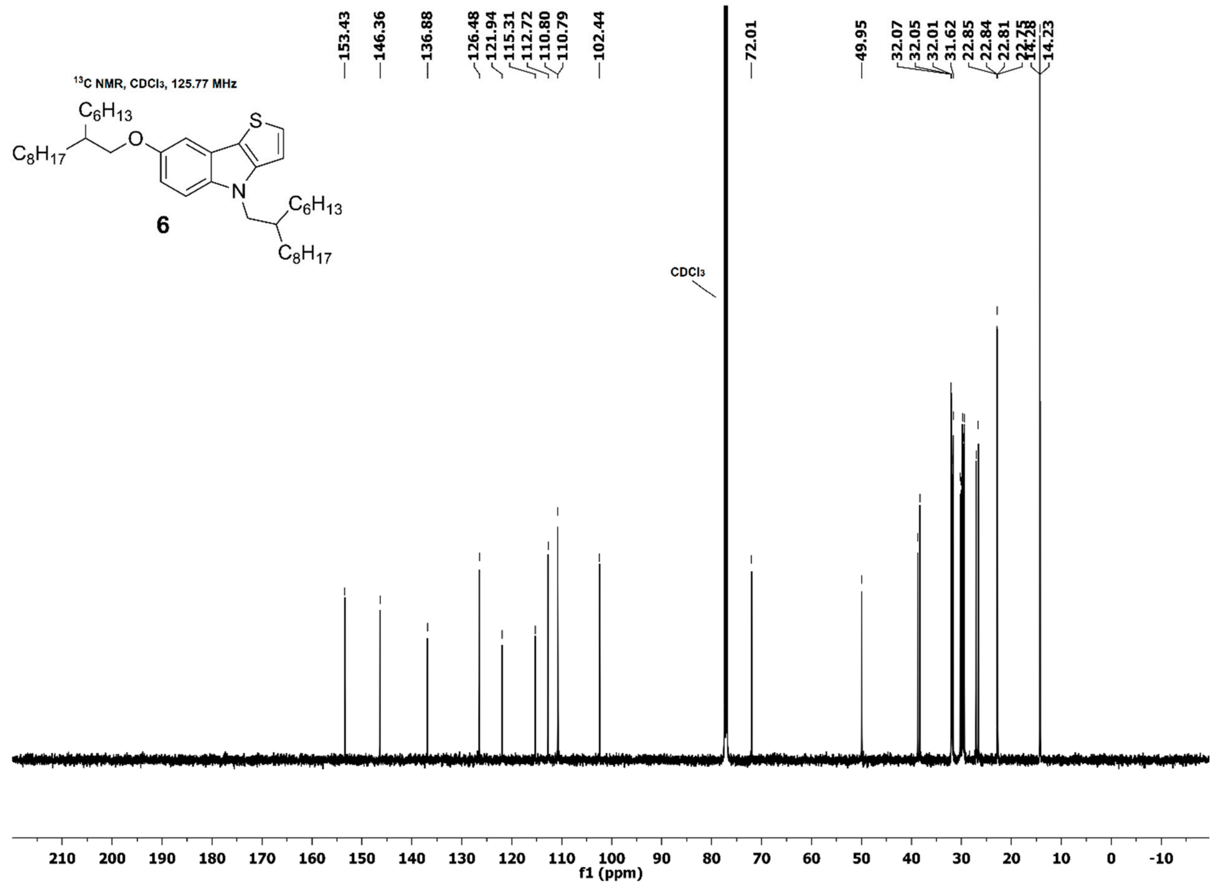

Figure S17. ^{13}C NMR spectrum of **6** ($CDCl_3$, 125.77 MHz).

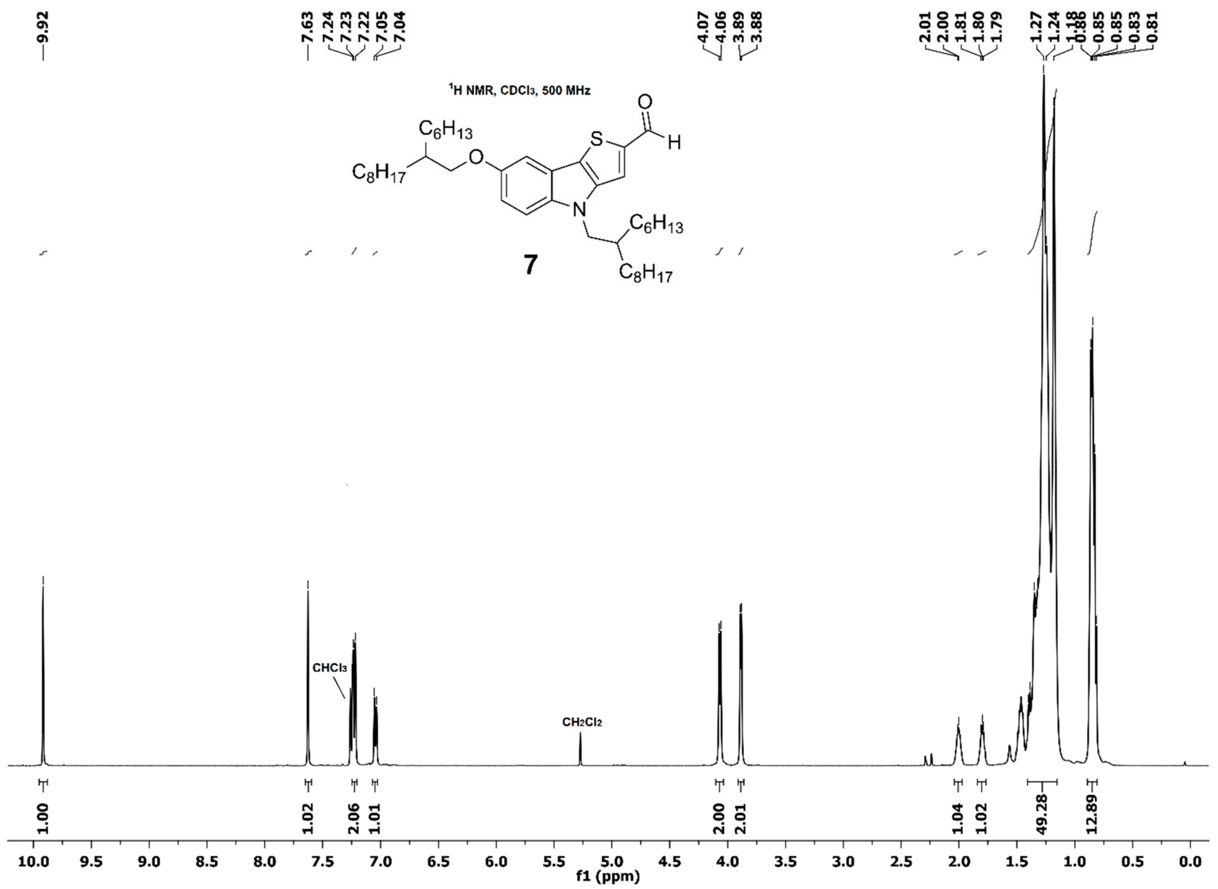

Figure S18. ^{1}H NMR spectrum of **7** ($CDCl_3$, 500 MHz).

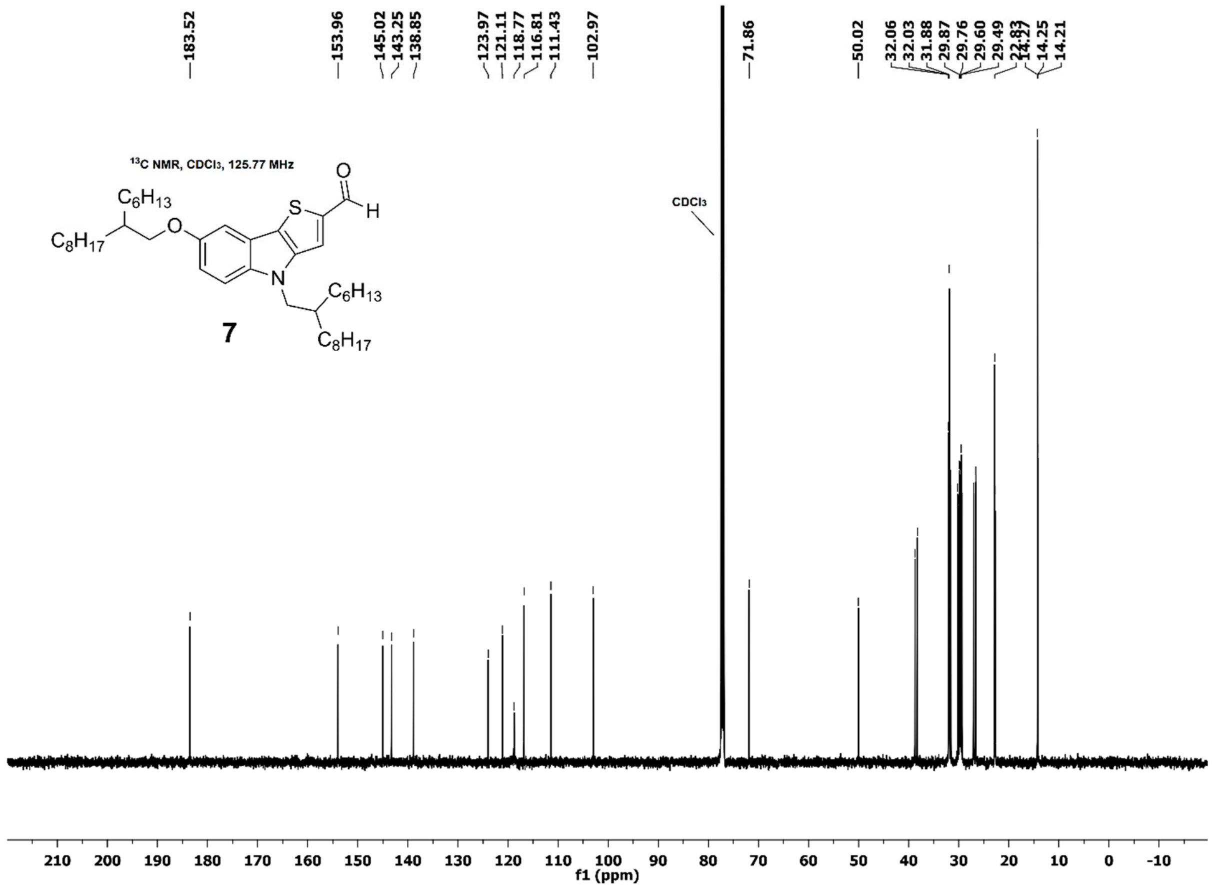

Figure S19. ^{13}C NMR spectrum of **7** ($CDCl_3$, 125.77 MHz).

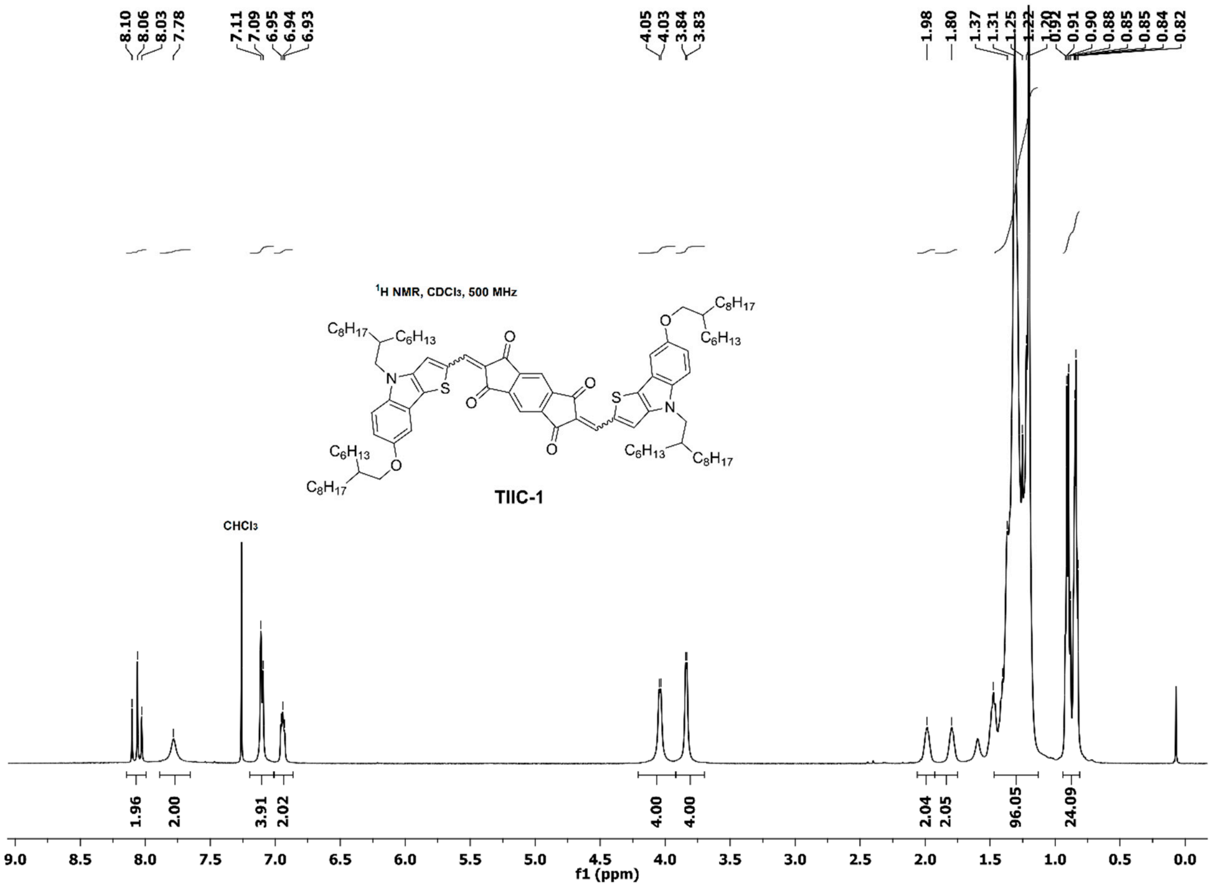

Figure S20. ^{1}H NMR spectrum of **TIIC-1** ($CDCl_3$, 500 MHz).

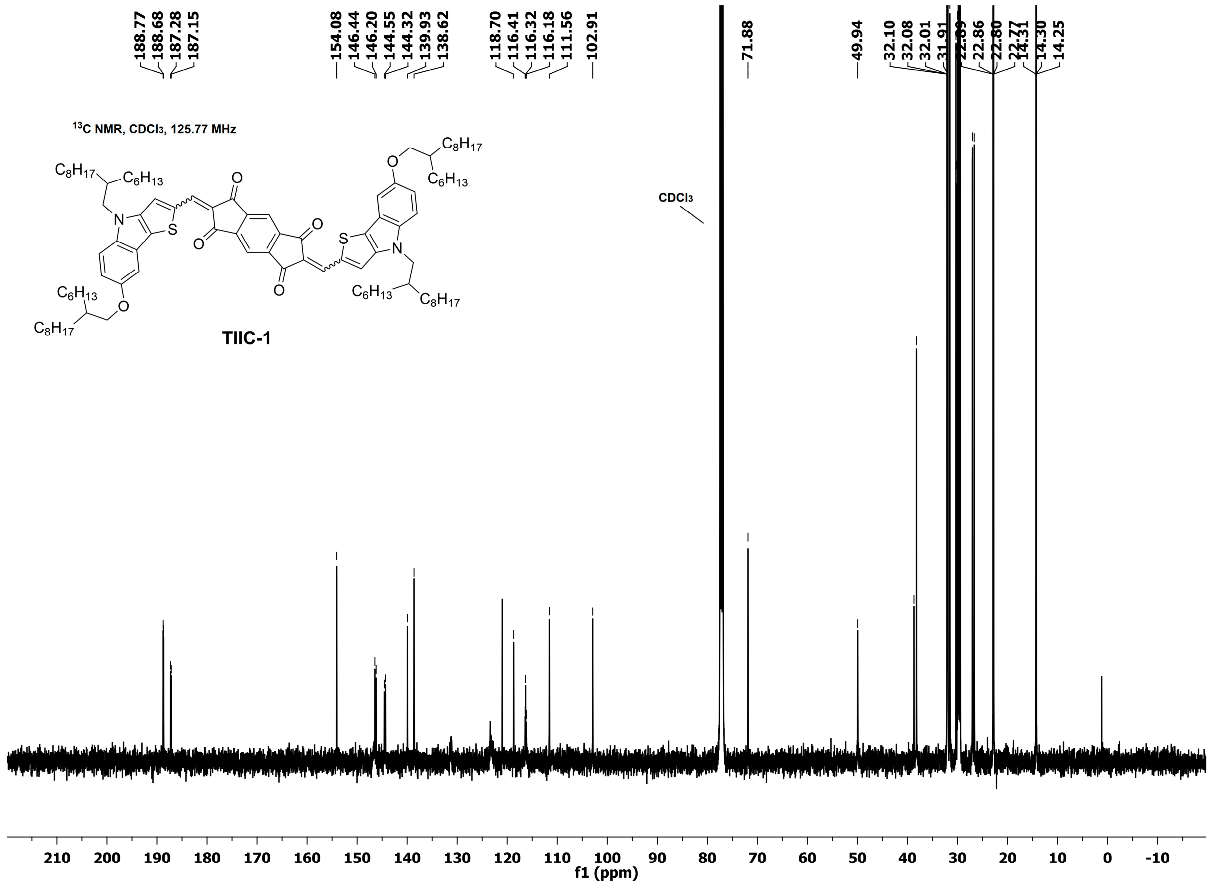

Figure S21. ^{13}C NMR spectrum of **TIIC-1** ($CDCl_3$, 125.77 MHz).

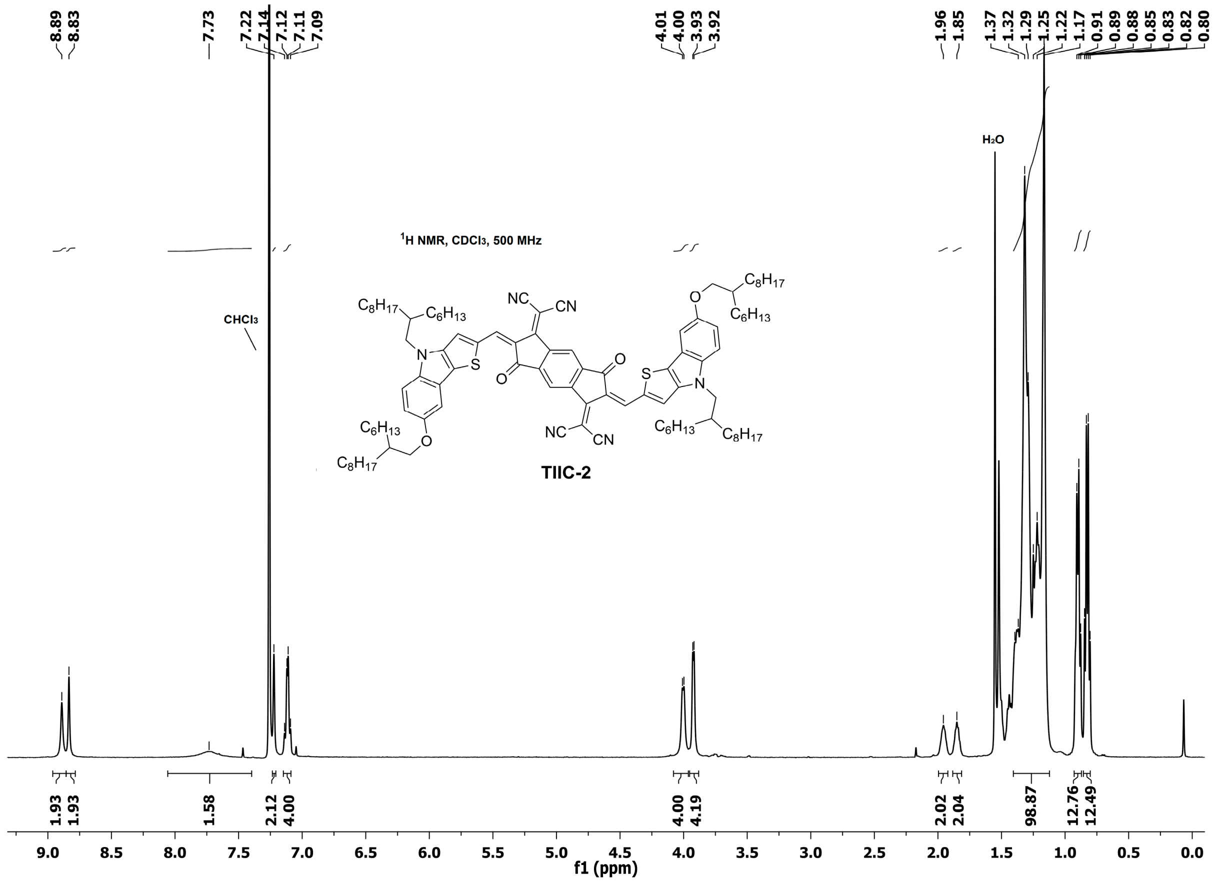

Figure S22. ^{1}H NMR spectrum of **TIIC-2** ($CDCl_3$, 500 MHz).

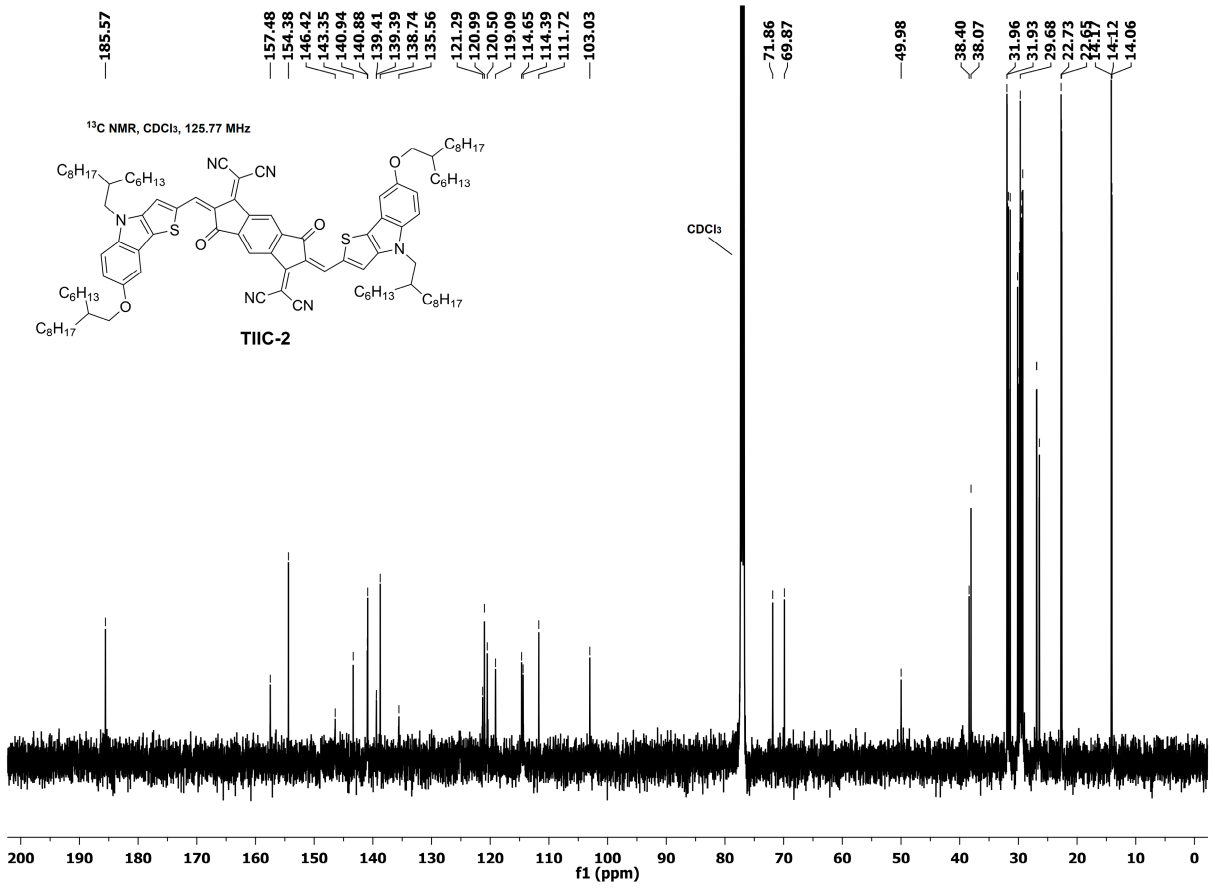

Figure S23. ^{13}C NMR spectrum of **TIIC-2** ($CDCl_3$, 125.77 MHz).
